# Pilot Alkaline Extraction of *Eucalyptus globulus* Bark: A Natural Sustainable Solution for Wood Preservation

**DOI:** 10.3390/antiox15060774

**Published:** 2026-06-22

**Authors:** Victor Ferrer, Tomás Oñate-Valdés, Cecilia Fuentealba, Gastón Bravo-Arrepol, Solange Torres, Vicente Hernández, Moisés Vásquez, Priscila Moraga-Suazo, Jorge Santos, Danilo Escobar-Avello

**Affiliations:** 1 Unidad de Desarrollo Tecnológico, Universidad de Concepción, Coronel 4191996, Chile; v.ferrer@udt.cl (V.F.); tonate@udec.cl (T.O.-V.); c.fuentealba@udt.cl (C.F.); 2 Centro Nacional de Excelencia para la Industria de la Madera (CENAMAD)—ANID BASAL FB210015, Pontificia Universidad Católica de Chile, Av. Vicuña Mackenna 4860, Santiago 7820436, Chile; vhernandezc@udec.cl (V.H.); priscila.moraga@uc.cl (P.M.-S.); 3 Facultad de Ciencias, Universidad San Sebastián, Lientur 1457, Campus Las Tres Pascualas, Concepción 4080871, Chile; gaston.bravo@uss.cl; 4 Laboratorio de Química de Productos Naturales, Departamento de Botánica, Facultad de Ciencias Naturales y Oceanográficas, Universidad de Concepción, Victor Lamas 1290, Concepción 4070386, Chile; soltorres@udec.cl; 5 Facultad de Medicina Veterinaria y Agronomía, Instituto de Ciencias Naturales, Universidad de las Américas, Campus El Boldal, Av. Jorge Alessandri 1160, Concepción 4090940, Chile; 6 Facultad de Ciencias Forestales/Centro de Biotecnología, Universidad de Concepción, Barrio Universitario s/n, Edificio Centro de Biotecnología, Concepción 4070386, Chile; movasquez2022@udec.cl; 7 Departamento de Ecosistemas y Medio Ambiente, Facultad de Agronomía e Ingeniería Forestal, Pontificia Universidad Católica de Chile, Campus San Joaquín, Av. Vicuña Mackenna 4860, Santiago 7820436, Chile; 8 ARCP-Associação Rede de Competência em Polímeros, 4200-355 Porto, Portugal; jorge.ucha@arcp.pt; 9 LEPABE-Faculty of Engineering, University of Porto, Rua Dr. Roberto Frias, s/n, 4200-465 Porto, Portugal; 10 ALiCE-Associate Laboratory in Chemical Engineering, Faculty of Engineering, University of Porto, Rua Dr. Roberto Frias, 4200-465 Porto, Portugal

**Keywords:** *Eucalyptus globulus* bark, alkaline extraction, wood preservation, polyphenolic extracts, antifungal activity, wood-decay fungi, UV weathering

## Abstract

In Chile, *Eucalyptus globulus* stands out as a significant forest species, yielding around 2 million tonnes of bark; this by-product is a valuable source of phenolic compounds. This research evaluated the valorization of *E. globulus* bark using alkali-assisted extraction (AAE) and obtained extracts intended to protect the wood against fungal degradation and ultraviolet (UV) radiation. The chemical and thermal properties of the extracts were characterized using total phenolic content (TPC), antioxidant capacity, FTIR spectroscopy, LC-LTQ-Orbitrap-MS, and thermal analyses (TGA and DSC). Pine wood samples were impregnated using the Bethel process, and their absorption, retention, leaching, UV resistance, gloss, and antifungal efficacy were evaluated. The AAE showed an extraction yield of 8.79%, almost double that of aqueous extraction, with a phenolic content of 970 mg GAE/100 g dry bark and good antioxidant capacity. The MS/MS analysis tentatively identified low-molecular-weight organic acids, phenolic acids, a hydrolyzable tannin derivative, ellagic acid, methylated flavonol glycosides, and an iridoid non-phenolic metabolite. Thermal analysis indicated greater stability of the alkaline extracts, with a mass loss of less than 10% up to 200 °C, and significant degradation between 220 and 300 °C. Leaching tests showed a lower release of polyphenols from alkali-treated wood, indicating reduced mobility and/or greater retention of the extractives within the wood structure. Biological assays demonstrated effective inhibition of stain fungi and strong resistance to brown rot. Furthermore, UV aging tests showed less color change (Delta E*) and greater resistance to surface degradation. These results demonstrate the potential of alkaline extracts from *E. globulus* bark as sustainable additives for wood protection.

## 1. Introduction

Wood is widely recognized as an excellent material thanks to its versatility, mechanical properties, and renewable nature. In sectors such as construction and furniture, wood is in high demand in regions such as Europe, Canada, and the United States [1,2]. However, in Chile, only around 17% of the construction sector uses timber, and there is a strong preference for alternative materials such as concrete [3]. The limited use of wood in residential construction is partly due to market and cultural perceptions of its durability, fire resistance, maintenance requirements, and perceived quality relative to more traditional materials such as concrete and masonry [4]. This highlights the need to develop strategies to reverse the trend and establish wood as a competitive and sustainable option for residential construction.

The organic nature of wood is one of its main limitations, making it susceptible to degradation by various abiotic factors, such as solar radiation and moisture, as well as biotic agents, including stain fungi, rot fungi, and termites. These factors all compromise the mechanical and physical properties of wood throughout its service life [5]. Wood degrades over time, particularly when exposed to outdoor weather conditions [6]. Under environmental conditions, wood degrades due to the action of light (both ultraviolet and visible radiation), which causes photochemical degradation of its main structural polymers, such as lignin, cellulose, and hemicellulose [7,8,9]. Of these components, lignin is the most sensitive to ultraviolet radiation, with a UV absorption coefficient of 80–95%, which is considerably higher than that of carbohydrates (5–20%) and extracts (approximately 2%) [6,8,9]. Other environmental factors contributing to wood degradation include temperature [10], water (i.e., moisture), wind carrying complex mixtures of suspended solid and liquid particles, and atmospheric pollution, as well as oxygen and ozone [11].

In terms of biotic degradation, three main groups of fungi that cause wood decay have been identified: brown rot, white rot, and soft rot. These organisms break down the structural polymers in wood cell walls, resulting in a significant loss of mechanical strength [7]. Wood can also be affected by molds and staining fungi, resulting in undesirable discoloration and reducing its commercial value [7]. Other biological agents capable of colonizing wood include bacteria, encrusting algae, and insects such as termites and beetles [6]. Consequently, developing products that protect wood by improving its durability, aesthetic quality, and mechanical strength is a daily challenge in scientific research and industrial practice.

Today, a wide range of strategies are used to protect wood against biotic and abiotic degradation, including impregnation processes, immersion treatments, and surface coatings such as stains, paints, and varnishes. These treatments are based on biological, enzymatic, chemical, or thermochemical principles. However, at an industrial level, chemical and thermochemical methods are primarily employed [6,8,12]. Methods of chemical surface modification for wood protection include acetylation, treatment with 1,3-dimethylol-4,5-dihydroxyethyleneurea (DMDHEU), furfurylation, and impregnation with thermosetting resins or vinyl monomers, among others [12]. Another widely used protection strategy, particularly against fungal degradation, is the impregnation with chemical preservatives, such as copper-based systems (CCA), triazoles, or boron-based fungicides [7,13,14]. While these treatments are effective, they are not environmentally friendly as they can leach out, leading to the accumulation of metal and metalloids and causing environmental impacts and potential risks to human health, including carcinogenic effects [7,15]. Furthermore, wood treated with metal-containing preservatives is unsuitable for combustion, composting, or recycling, which compromises the sustainability of the resource. Furthermore, some treated wood products must be classified as hazardous waste due to the gradual release of metals into soil and water bodies at levels that pose contamination risks [15].

In recent years, wood protection strategies based on natural compounds have attracted growing scientific and technological interest due to their environmental friendliness. These strategies use plant and wood extracts, essential oils, waxes, bark resin, and biopolymers, all of which are generally considered to have a low environmental impact. However, their practical applications are currently limited by their tendency to leach from the wood structure during use [8]. Among these alternatives, natural phenols and polyphenols are particularly promising for wood protection. These compounds are widely present in plants, playing important roles in chemical defense, pigmentation, structural reinforcement, and protection against ultraviolet radiation [7,16,17,18,19,20]. This diverse group includes epicatechin, epigallocatechin gallates, ellagic and tannic acids, among others [16,21,22]. In general, plant-derived polyphenols exhibit a wide range of physical and chemical properties, conferring high chemical versatility, including UV absorption, radical-scavenging activity, and metal-ion chelation [16,21]. For instance, promising results have been reported for polyphenol-rich bark extracts applied to synthetic resin systems to enhance wood’s resistance to solar radiation and outdoor exposure [23,24]. Similarly, Mishra et al. [25] evaluated the antifungal activity of a heartwood extract from *Eucalyptus bosistoana* against the brown-rot fungus *Coniophora cerebella* Pers., concluding that the observed high bioactivity was associated with the presence of hexadecanoic acid. In this regard, multiple studies indicate growing interest in using natural extracts for environmentally friendly wood preservation [17,26,27].

However, the practical application of natural extracts for wood protection is still limited by their tendency to leach from the wood during use [28]. This is mainly due to the presence of low-molecular-weight phenolic compounds and highly water-soluble tannin fractions, which are easily mobilized in wet conditions [29,30]. Therefore, it is important to study extraction strategies that influence the chemical composition and leaching behavior of these extracts to develop more durable bio-based wood protection systems.

Eucalyptus bark extracts have been reported to be rich in bioactive compounds with potential pharmaceutical value, primarily due to their anti-inflammatory, antimicrobial, antibacterial, and antioxidant properties [31,32]. In line with this, recent studies by Santos et al. [33] showed that alkaline extract of eucalyptus bark (AEEB) contains high levels of quercetin- and isorhamnetin-derived compounds, which are well known for their antifungal properties [34,35]. However, the direct use of AEEB as an antifungal and UV-protective agent for wood protection remains scarcely explored. This is a particularly relevant issue in Chile, where eucalyptus bark is an abundant forestry by-product with an estimated annual production of around 1.5 million cubic meters. Currently, most of this material is valorized as boiler fuel in wood-processing facilities, while the fraction unsuitable for energy recovery is sent to landfills [36,37]. Therefore, developing new ways to utilize this by-product within the forestry sector could significantly contribute to the industry’s circular economy strategies.

This study investigated the valorization of *E. globulus* bark through a pilot-scale alkaline extraction process to obtain a bio-based additive for wood preservation. To the best of our knowledge, no previous studies have evaluated the impregnation treatment using AEEB as a dual-function strategy for both antifungal and anti-UV protection. The research hypothesis was that a low-concentration alkali-assisted extraction process can recover phenolic-rich fractions from *E. globulus* bark that, after wood impregnation, may reduce extractive mobility and improve resistance to UV-induced weathering and fungal degradation. To test this hypothesis, the specific aims were: (i) to produce an alkaline extract from *E. globulus* bark at pilot scale; (ii) to characterize its phenolic content, antioxidant capacity, chemical profile, functional groups, and thermal behavior; (iii) to impregnate pinewood samples with the alkaline extract under controlled conditions; and (iv) to evaluate the leaching behavior, UV-weathering performance, and biological resistance of the treated wood against staining and decay fungi.

## 2. Materials and Methods

### 2.1. Raw Material

Eucalyptus bark (*E. globulus*) was obtained from Forestal Collicura in Santa Juana, Bio-Bio region, Chile. The bark was first screened through a 20 mm mesh as a preliminary cleaning step to remove oversized materials, such as sticks, branches, and large wood chips. It was then ground using a Breuer M8 hammer mill (BTD, St. Vith, Belgium). The resulting fibrous material was subsequently sieved to 4 mm to remove dust and fine particles. The composition of the *E. globulus* bark (expressed as weight percent on an oven-dry basis) was cellulose 49.36%, hemicellulose 15.70%, acids 4.73%, total lignin 17.82%, and acetone extractables 2.16%. This compositional analysis was performed as an external analytical service by the Instituto de Investigaciones Tecnológicas of the Universidad de Concepción (IIT-UdeC, Concepción, Chile). Briefly, extractable compounds were determined using 90% acetone. The extracted material was then subjected to acid hydrolysis to quantify insoluble lignin (Klason lignin), soluble lignin by spectroscopic analysis, and carbohydrates by HPLC, as reported by the service provider.

### 2.2. Pilot Alkaline Extraction (AAE)

**Figure 1:**
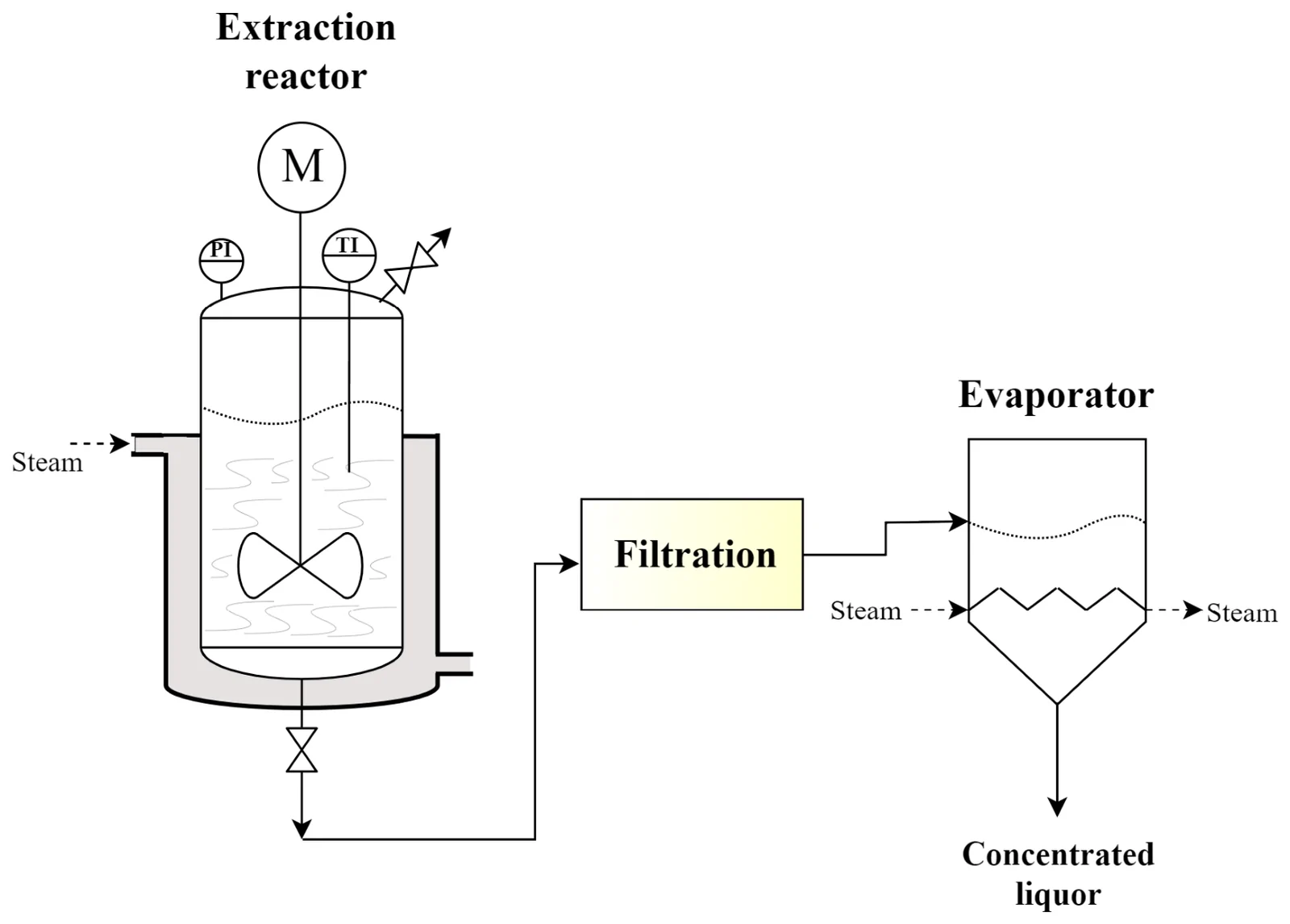
Diagram of the eucalyptus bark extraction process.

The extraction of eucalyptus bark was carried out in a 100 L stainless steel jacketed reactor equipped with a mechanical stirring under the following conditions: temperature: 80 °C; time: 100 min; substrate to 0.05% *w*/*v* NaOH (Winkler, 97%, Santiago, Chile) solution ratio 1:30 *w*/*v*. A total of 2.8125 kg of *E. globulus* bark, expressed on an oven-dry basis, was loaded into the reactor together with 84.375 L of alkaline solution. The bark was used as received after screening and milling, with an initial moisture content of 10.5%, and was not subjected to any prior oven drying or thermal treatment. NaOH solution was selected as a mild alkaline extraction aid to improve the release of phenolic compounds from the bark matrix, based on previous studies reporting bioactive flavonoid-derived compounds in alkaline eucalyptus bark extracts [33,38,39]. The pH of the NaOH solution was 12.8. At the end of the extraction period, the liquid extract was drained from the reactor bottom and passed through a 100 µm nylon filter bag to separate bark residues from the liquor. The pH of the resulting liquor was around 7.5. Finally, the liquor was concentrated to 2.5% *w*/*w* using an evaporator (Artur Probst, Leverkusen, Germany) at an evaporation rate of 120 L/h. A diagram of the extraction process is shown in Figure 1.

The yield of the extraction (%*Y*) was determined using Equation (1):

(1) $$Y(\%)=\frac{mass\;extracted\;liquor\left(g\right)\times solids\;in\;liquor\;(\%\frac{w}{w})}{mass\;of\;dried\;feed\;sample}$$

The solids in the liquor were determined by oven-drying aliquots at 105 °C overnight until constant weight; measurements were performed in triplicate. The extract obtained by alkali-assisted extraction in the 100 L reactor was coded as AE100L. The alkaline and water extracts obtained in the 6 L reactor, used for comparison, were coded as AE6L and WE6L, respectively.

### 2.3. Characterization of the Extract

#### 2.3.1. Determination of Total Phenolic Compound (TPC)

The Folin–Ciocalteu (F-C) spectrophotometric method adapted to 96-well microplates was used to determine the TPC of the prepared extract solutions [40]. Each sample (24 μL) was mixed with 184 μL of Milli-Q water. In addition, 12 μL F-C reagent (2N) and 30 μL sodium carbonate (20%, *w*/*v*) were added. After mixing, the solutions were incubated in the dark at room temperature (~15 °C) for 60 min. The absorbance of the samples was measured at 750 nm using a spectrophotometer (SPECTROstar nano, BMG LABTECH, Ortenberg, Germany) that was used for all antioxidant assays. A calibration curve was established using gallic acid as the standard over a concentration range of 10–200 mg/L. Total phenolic compounds (TPC) were expressed as milligrams of gallic acid equivalent per 100 g of dry bark (mg GAE/100 g db). All assays were performed in triplicate.

#### 2.3.2. Determination of Antioxidant Capacity (ABTS, DPPH, FRAP)

The antioxidant capacity using ABTS (2,2′-Azino-bis (3-ethylbenzthiazoline-6-sulfonic acid), DPPH (2,2-diphenyl-1-picrylhydrazyl), and FRAP (ferric ion reducing antioxidant potential) methods was completed in 96-well microplates using a microplate reader (SPECTROstar nano BMG LABTECH, Ortenberg, Germany).

The ABTS assay was conducted as described in Escobar-Avello et al. [41] and evaluates the extract’s ability to inhibit the oxidation of ABTS free radicals. Briefly, an ABTS radical cation solution (7.5 mM) with K_2_S_2_O_8_ (2.5 mM) was diluted with ethanol to obtain an absorbance of 0.7 at 734 nm. Then, 190 μL of diluted ABTS^•+^ radical solution was added to each well. After incubation for 5 min at 30 °C, the initial absorbance was recorded at 734 nm. Subsequently, a 10 μL aliquot of either the sample or the Trolox standard was added, and the plate was incubated at 30 °C for 20 min.

For the DPPH assay, which measures the extract’s free radical-scavenging potential, a 0.1 mM DPPH solution was prepared in 80% (*v*/*v*) ethanol/water. Then, 25 μL of the sample and 250 μL of the DPPH solution (1:10 ratio) were mixed in a 96-well microplate. After shaking, the mixture was incubated in the dark at room temperature for 60 min. After the reaction period, the samples’ absorbance was measured at 515 nm using a spectrophotometer with a microplate reader. The calibration curve was prepared using gallic acid as the standard, with concentrations ranging from 1 to 40 mg/L.

The FRAP assay was performed according to the methodology indicated by Sricharoen et al. [42]. The FRAP method assesses the capacity of antioxidants to reduce the ferric-tripyridyl-triazine (Fe^3+^-TPTZ) complex to its blue-colored ferrous form, which absorbs light at 593 nm. A buffer solution at pH 3.6 was prepared using sodium acetate and acetic acid at a concentration of 300 mM. Then, a 10 mM TPTZ (2,4,6-tris(2-pyridyl)-s-triazine) solution in 40 mM hydrochloric acid was combined with a 20 mM iron chloride (FeCl_3_) solution. The FRAP working solution was obtained by mixing the buffer, TPTZ, and FeCl_3_ in a 10:1:1 (*v*/*v*/*v*) ratio. Then, 250 μL of this working solution was mixed with 25 μL of the sample or standard in a 1:10 (*v*/*v*/*v*) ratio in a 96-well microplate. The mixture was shaken and incubated at 37 °C for 6 min to allow the reaction to proceed. The calibration curve was generated using gallic acid as a standard, with concentrations ranging from 1 to 200 mg/L.

For all assays, antioxidant capacity was expressed as milligrams of gallic acid equivalents per 100 g of dry bark (mgGAE/100 g db), and all measurements were performed in triplicate (n = 3).

#### 2.3.3. Fourier Transform Infrared Spectroscopy (FTIR) Assay

To identify the molecular functional groups of the bioactive compounds present in the Eucalyptus bark extract, FTIR absorption spectra of the powdered extracts were performed on a BRUKER Alpha T FTIR spectrophotometer (Bruker Corporation, Billerica, MA, USA) equipped with an attenuated total reflectance (ATR) unit. Spectra were obtained using 64 scans from 4000 to 400 cm^−1^ at a resolution of 4 cm^−1^. These scans were processed using OPUS 7.0 software.

#### 2.3.4. LC-LTQ-Orbitrap-MS Analyses

The phenolic composition of the alkaline extract was analyzed using liquid chromatography coupled to high-resolution mass spectrometry on an Accela LC system (Thermo Fisher Scientific, Hemel Hempstead, UK), which was equipped with a diode array detector, a thermostated autosampler, and a quaternary pump. Separation was performed on an Atlantis T3 reversed-phase C18 column with a silica-based, trifunctionally bonded C18 alkyl stationary phase and T3 end-capping technology (3 µm, 2.1 mm × 100 mm; Waters Corporation, Milford, MA, USA). The mobile phase consisted of solvent A (water acidified with 0.1% formic acid) and solvent B (CH_3_OH). Elution was carried out at a flow rate of 0.35 mL/min with the following gradient: 2% B at 0 min, 8% B from 0 to 2 min, 20% B from 2 to 12 min, 30% B from 12 to 13 min, and 100% B from 13 to 17 min. The gradient was reset to 2% B between 17 and 18 min, followed by a 5 min re-equilibration period under initial conditions. The injection volume was 5 µL [40].

The liquid chromatography system was coupled to an LTQ-Orbitrap XL mass spectrometer (Thermo Fisher Scientific, Hemel Hempstead, UK), which was equipped with an electrospray ionization source operating in negative ion mode. Data acquisition and instrument control were performed using Xcalibur 3.0 software. Spectra were recorded in FTMS mode within the *m*/*z* range of 120 to 2000. Full-scan MS spectra were acquired at a resolution of 60,000 full width at half maximum (FWHM) at *m*/*z* 400, while data-dependent MS/MS experiments were obtained at a resolution of 15,000. The most abundant precursor ions detected in the full-scan spectrum were automatically selected for fragmentation. Fragmentation was performed using high-energy collision dissociation with a normalized collision energy of 35% and an activation time of 20 ms. The ion source parameters were set as follows: capillary temperature, 350 °C; sweep gas, 1 arbitrary units (a.u.); auxiliary gas, 10 a.u., and sheath gas, 50 a.u. Mass accuracy was maintained below 5 ppm. Compounds were tentatively identified by combining exact mass data with MS/MS fragmentation patterns and comparing them with those reported in the literature [43,44].

#### 2.3.5. Thermogravimetric Analysis (TGA)

To evaluate the thermal stability and decomposition behavior, TGA of the powdered extracts was performed using a TG 209 F3 Tarsus instrument (NETZSCH, Selb, Germany). Samples weighing between 2 and 5 mg were placed in aluminum crucibles and heated from 25 to 600 °C at a rate of 10 °C/min under a nitrogen atmosphere (AGA, 99.995%). The TGA data were presented as percentage weight loss and its first derivative over time.

#### 2.3.6. Differential Scanning Calorimetry (DSC)

To identify thermal transitions and heat flow associated with physical or chemical changes, DSC was conducted using a NETZSCH 204 F1 Phoenix instrument (NETZSCH, Selb, Germany). Samples weighing between 1 and 2 mg were placed in aluminum pans with pierced lids and heated from 25 °C to 300 °C at 10 °C/min under a nitrogen purge of 20 mL/min.

### 2.4. Impregnation of Wood

Pinewood specimens (19 mm (longitudinal) × 19 mm (tangential) × 19 mm (radial) for leaching and fungal tests, and 100 (longitudinal) × 50 (tangential) × 19 mm (radial) for aging chamber tests) with an average density of 0.467 g/cm^3^, were supplied by the Arauco Company in Chile’s Bio-Bio region. The Pinewood materials were previously oven-dried for 24 h at 105 °C and then weighed before impregnation. Subsequently, they were impregnated with the prepared extracts, following the vacuum–pressure–vacuum full-cell method [45]. A vacuum of −0.7 bar was applied for 15 min, after which the liquid to be impregnated was introduced under the vacuum. Once the liquid was introduced, a pressure of 6 bars was applied for 1 h. The liquid was removed at the end of the impregnation time, and a new vacuum stage was performed for 15 min. Finally, the excess liquid on the specimens’ surfaces was removed, and their weight was recorded after impregnation. The impregnated samples were stored at room temperature for at least 72 h before testing.

The determination of Absorption (*A*) was performed using the following Equation (2):

(2) $$A=\frac{m_{2}-m_{1}}{V}$$

where

*A*: Absorption, kg/m^3^.

*m*_1_: Weight of the specimen before treatment, kg.

*m*_2_: Weight of specimen after treatment, kg.

*V*: Volume of the specimen, m^3^.

Retention (*R*) was determined from Equation (3):

(3) $$R=\frac{m_{2}-m_{1}}{V}\times \frac{C}{100}$$

where:

*R*: Retention, kg/m^3^.

*C*: Concentration of preservative, %.

To express the amount of retained extract in the wood as a percentage of the weight gained after impregnation, (%*REW*) was calculated from Equation (4):

(4) $$\%REW=\frac{R}{A}\times 100$$

### 2.5. Accelerated UV Weathering and Color Measurement

Impregnated and control pinewood specimens were exposed to UV radiation at 340 nm in a QUV accelerated weathering tester (Q-LAB Corporation, Westlake, OH, USA) for 1000 h, following cycle 7 of ASTM G154 [46]. A commercial product (CP sample, stain-type penetrating wood protector, Cerestain by Ceresita^®^, Santiago, Chile), a NaOH solution, and water were used as control treatments. The color of wood samples exposed to artificial UV radiation was measured at the beginning of the assay and after the total exposure. Color parameters expressed in CIE Lab* coordinates were recorded using a BIOBASE BCM-200 colorimeter (BIOBASE, Jinan, China). The color difference (Delta E*) was determined for each sample by comparing the color values before and after exposure, following Equation (5):

(5) $$Delta\;E^{*}={\left[{\left({L^{*}}_{2}-{L^{*}}_{1}\right)}^{2}+{\left({a^{*}}_{2}-{a^{*}}_{1}\right)}^{2}+{\left({b^{*}}_{2}-{b^{*}}_{1}\right)}^{2}\right]}^{\frac{1}{2}}$$

where L* represents lightness, a* corresponds to the green-red axis, and b* to the yellow-blue axis. The subscripts (1) and (2) indicate measurements taken before and after exposure. For each sample, measurements were taken at four randomly selected positions on the tangential surface, and the mean was calculated.

The appearance of cracks in the wood was evaluated both visually and photographically, by analyzing photos taken every 50 h of exposure in the accelerated weathering tester (Q-LAB, QUV, USA), up to a total of 1000 h.

The gloss of the painted samples was measured before and after the test using a gloss meter (Shenzhen 3nh Technology Co., Model YG268, Shenzhen, China). An average of three random measurements was taken, with the angle of incidence set to 60° on the tangential surface. The gloss value before (*G*_0_, 12 h control) and after the test (*G_f_*) was reported, and the gloss retention ratio was calculated according to Equation (6):

(6) $$Gloss\;retention\;ratio\;(\%)=\frac{G_{f}}{G_{0}}\times 100\;$$

### 2.6. Leaching Test

The leaching test was performed according to EN 84:2020 “Wood preservatives—Accelerated ageing of treated wood prior to biological testing—Leaching procedure” [47]. Six wooden cubes were taken for each extract type (previously dried at 105 °C for 24 h and weighed, *m*_0_) and placed in beakers, to which distilled water was added at a 1:5 ratio to the sample volume (205 mL of distilled water per beaker). The water was then changed nine times over 14 days. The leaching liquid samples were stored for subsequent TPC analysis at each time. After the test, the samples were dried at 105 °C for 24 h and weighed. The percentage weight loss (*WL*%) was determined using Equation (7):

(7) $$WL(\%)=\frac{m_{0}-m_{1}}{m_{0}}\times 100$$

where:

*m*_0_: Weight of the test piece before leaching, g.

*m*_1_: Weight of the test piece after leaching, g.

### 2.7. Antifungal Tests

#### 2.7.1. Staining Fungi

The standard fungicide test method was used to control sapwood stain and mould on undried wood (ASTM D4445-10) [48]. The stain fungi *Trichoderma viride* and *Diplodia seriata* are strains available in the Wood Technology laboratory of the Faculty of Forest Sciences, University of Concepcion, and used to obtain spores and mycelium, respectively, from in vitro cultures. As *T. viride* and *D. seriata* have different sporulation behaviors, they were inoculated using conidial (spore) and mycelial (hyphal) suspensions, respectively. Mycelium inoculation provides a more aggressive and reproducible challenge for *D. seriata* than a conidial suspension [49]. The inocula were not intended to be quantitatively equivalent between species, and the inhibition values were interpreted on an intra-species basis. To ensure uniform inoculum density among treatments within each fungus, each suspension was homogenized and applied at a fixed volume (approximately 0.25 mL per specimen) so that treated specimens, negative controls, and positive controls received the same inoculum load. For culture preparation, the fungi were grown on malt extract agar (2%) and incubated at 20 °C for 10 days. *T. viride* spores and *D. seriata* mycelium were obtained in a sterile saline solution (0.9% NaCl). Pinewood specimens (7 × 20 mm cross-section and 70 mm length) were autoclaved at 121 °C for 20 min. The specimens were immersed in the solution for 10 s per treatment, then aerated in a sterile environment for 24 h before inoculation with fungi. The Nipacide fungicide was used as a positive control, prepared as a 5% *v*/*v* aqueous solution. After drying, the wooden specimens were placed in sterile Petri dishes (five replicates per treatment). To maintain high humidity during the incubation, absorbent filter paper was placed at the bottom of each Petri dish and moistened with 1 mL of sterile distilled water. A polyethylene mesh spacer was placed over the paper to avoid direct contact between the wood specimens and the wet surface. The *T. viride* conidia suspension and the *D. seriata* mycelium suspension were gently shaken during the inoculation procedure to ensure homogeneity. Approximately 0.25 mL of each suspension was applied along one side of each wood sample in the Petri dish to ensure comparable inoculum density across all treatments. Additionally, a small amount of the inoculum was placed on one of the transverse ends of the sample. After inoculation, the Petri dishes were sealed with Parafilm^®^ to prevent moisture loss and incubated in the dark at 25 °C. The incubation period was 4 weeks, with monitoring every 7 days. When necessary, sterile distilled water was added to maintain moisture conditions. After the incubation period, fungal growth was visually assessed and scored on a scale of 0 to 5, with 5 representing the highest degree of fungal development. The evaluation considered both growth intensity and surface discoloration, excluding localized spots attributable solely to initial inoculum placement rather than to active fungal colonization. The percentage inhibition of staining fungi was calculated using Equation (8):

(8) $$Inhibition(\%)=1-\frac{sample\;value}{control\;value}$$

where the sample value represents the average of the values obtained from the treated specimens at the end of the test, whereas the control value is calculated from the average of the untreated specimens at the end of the test. The water-treated specimens served as the negative control group for fungal inhibition calculations.

#### 2.7.2. Decay Test

The decay test was conducted in accordance with AWPA E10-16 [50], using the brown-rot fungus *Postia placenta* and the soil-block test method. Glass jars (500 mL) were half-filled with peat to prepare the decay chambers, and the moisture content was adjusted to approximately 70% by adding distilled water. Pre-moistened feeder strips (3 mm × 20 mm × 20 mm) of pine sapwood were placed on the soil surface. The jars were then sterilized in an autoclave at 121 °C for 1 h. After cooling, fungal cultures were added to the feeder strips and incubated at 28 °C until the mycelium had completely covered the wood surface. Wood samples (19 mm × 19 mm × 19 mm) were oven-dried at 60 °C to remove moisture and weighed (*m*_0_, see Equation (6)). They were then sterilized in an autoclave and immersed in distilled water to achieve the necessary moisture content for fungal colonization. Following this, *Pinus radiata* samples (19 mm × 19 mm × 19 mm) were placed on the feeder strips and incubated at 28 °C and 75% relative humidity for 12 weeks. Four replicates per treatment were exposed to fungi. After incubation, all samples were removed from the glass jars, and any surface mycelium was carefully removed. The samples were then oven-dried at 60 °C, and their final dry weight (*m*_1_, see Equation (7)) was recorded. The extent of decay was assessed by calculating the percentage weight loss due to decay (*WLD*%) using the following Equation (9):

(9) $$WLD(\%)=\frac{m_{2}-m_{3}}{m_{2}}\times 100$$

where *m*_2_ represents the initial dry weight of the wood sample before exposure, and *m*_3_ corresponds to the dry weight after 12 weeks of fungal attack.

### 2.8. Statistical Analysis

The results were presented as the mean ± standard deviation (SD) of independent measurements. Prior to statistical comparison, the normality assumption was assessed using the Shapiro–Wilk test. For datasets with a limited number of observations per treatment, normality was evaluated using the residuals from the ANOVA model. One-way analysis of variance (ANOVA) was performed to assess statistical significance, followed by Tukey’s post hoc test. A *p*-value ≤ 0.05 was considered statistically significant.

## 3. Results and Discussion

### 3.1. Impact of Extraction Conditions on the Yield of Eucalyptus Bark Extraction

Table 1 reports the yield obtained for the AAE performed in the 100 L reactor (AE100L). For comparative purposes, the results of AE6L and WE6L extractions are included. These extractions were performed under the same conditions used for the extraction in the 100 L reactor. It has been widely reported that the use of alkali for bark extraction increases the yield of tannins as well as other components present in the bark [33,38,51,52,53]. An alkaline solution promotes increased leaching due to diffusion and cell wall breakdown [39]. Vásquez et al. [38] performed eucalyptus bark extraction trials using basic NaOH, Na_2_SO_3_, and Na_2_CO_3_ solutions, obtaining a higher yield (between 9.81 and 18.9%) when NaOH was used in concentrations between 2.5 and 10%. The lower observed yield (8.79%) is likely due to the low NaOH concentration (0.05% *w*/*v*), which was selected to reduce environmental impact and neutralization demand. Higher NaOH concentrations would likely increase the extraction yield, as reported for eucalyptus bark extracted under stronger alkaline conditions [38]. However, they may also promote the co-extraction of non-target bark components and produce highly alkaline liquors that require pH adjustment before wood impregnation. In general, extraction yields are highly influenced by temperature, time, and solvent type [54]. Although the solids concentration in the liquor was lower for sample AE100L than for AE6L, the higher extraction yield for AE100L was due to the greater amount of liquor recovered per unit of dry bark. The ratio of extracted liquor mass to dry feed mass was 28.1 g/g for AE100L and 17.0 g/g for AE6L. This resulted in a greater total mass of solids being extracted in the pilot-scale reactor. This improvement was likely due to more efficient liquid-solid contact and liquor recovery, which was facilitated by the mechanical stirring system in the 100 L reactor. In contrast, the 6 L reactor relied primarily on recirculation.

Compared with water extraction, alkali-assisted extraction increased the yield, probably due to the alkaline swelling of the bark matrix and enhanced solubilization of phenolic compounds and other alkali-soluble components, as previously reported for alkaline extraction of bark biomass [55]. One aspect to note is that, although the pH of the extraction solution is basic (between 12 and 13), the pH of the extract at the end of the alkaline extraction is around 7–8. This pH reduction is attributed to the acidic nature of the extracted phenolic compounds, which act as buffering agents.

### 3.2. LC-LTQ-Orbitrap-MS

The chemical profile of the alkaline extract of *E. globulus* bark was characterized using LC-ESI-LTQ-Orbitrap-MS in negative ionization mode. Table 2 lists compounds tentatively identified from the chromatogram (see Figure S1), including low-molecular-weight organic acids, phenolic acids, hydrolyzable tannin derivatives, ellagic acid-related compounds, an iridoid derivative, and methylated flavonol glycosides. Although several of these compound families have been previously reported in *E. globulus* bark extracts [32,56], their detection in the present study confirms that the mild pilot-scale alkali-assisted extraction process used here recovered key phenolic and tannin-related constituents. Therefore, the LC-LTQ-Orbitrap-MS analysis provides chemical support for the subsequent evaluation of this extract as a bio-based additive for wood impregnation and preservation.

Low-molecular-weight organic and phenolic acids were identified. Malic acid was detected at Rt of 1.78 min, whose fragmentation pattern is consistent with that reported for malic acid in negative ionization mode [57].

Quinic acid (Rt = 2.07 min) was detected with a precursor signal at *m*/*z* 191.0195. The MS/MS spectrum showed characteristic fragments at *m*/*z* 173 (loss of H_2_O), 111, 87, and 85, which are consistent with the fragmentation patterns reported for this compound in Eucalyptus species [32]. Gallic acid was identified at Rt = 4.45 min, with its main fragment at *m*/*z* 125 corresponding to the loss of a CO_2_ molecule [32,40,58]. The presence of gallic acid, a key structural unit associated with gallotannins [56], suggests the presence of galloylated phenolic structures, or their partial hydrolysis, in the alkaline extract. Similarly, the presence of protocatechuic acid was observed at 10.40 min and identified by its fragments at *m*/*z* 109 and 91, which are also associated with decarboxylation [56,58]. The detection of gallic and protocatechuic acids is relevant because these compounds are commonly associated with galloylated structures and hydrolyzable tannins, including gallotannins, ellagitannins, and ellagic acid-derived compounds, which have been widely reported in Eucalyptus bark and other lignocellulosic tissues [32,62,63].

The alkaline extract also contained a high-molecular-weight hydrolyzable tannin derivative. The signal observed at Rt of 16.19 min was tentatively identified as casuarinin (galloyl-bis(hexahydroxydiphenoyl)-glucose). The MS/MS spectrum showed fragments that are characteristic of units related to ellagic and gallic acids [32].

The signal, detected at Rt of 16.58 min, was assigned to loganetin. This compound was tentatively identified based on its mass spectral features, which were consistent with those previously reported for extracts obtained from the leaves and branches of *E. globulus* [59]. Although loganetin is not a phenolic compound, its presence indicates that the alkaline extract contains other metabolites, thereby broadening the extract’s chemical profile beyond strictly polyphenolic constituents.

Ellagic acid was identified with a retention time of 18.06 min, and the obtained fragments are consistent with the sequential loss of a carbonyl group and decarboxylation of the ellagic acid structure. Ellagic acid is one of the most prevalent phenolic compounds found in the bark of *E. globulus* and is often linked to the hydrolysis or degradation of ellagitannins containing hexahydroxydiphenol (HHDP) [32,61]. The detection of this compound, alongside casuarinin, highlights the importance of hydrolyzable tannins and ellagitannin-derived structures in the alkaline extract’s chemical composition.

Finally, flavonoids belonging to the isorhamnetin family were identified. Signals corresponding to isorhamnetin-hexoside and -rhamnoside were detected at two different retention times: 17.71 min and 18.18 min [32,60]. The MS/MS spectra revealed the aglycone of isorhamnetin at *m*/*z* 315 and 314 after the loss of the hexose unit (162 Da) or its derivatives. The variety of these glycosylated derivatives indicates that the extract contains a substantial proportion of flavonoids, which contribute to its potential antioxidant capacity and thermal stability.

### 3.3. FTIR Spectroscopy Analysis

**Figure 2:**
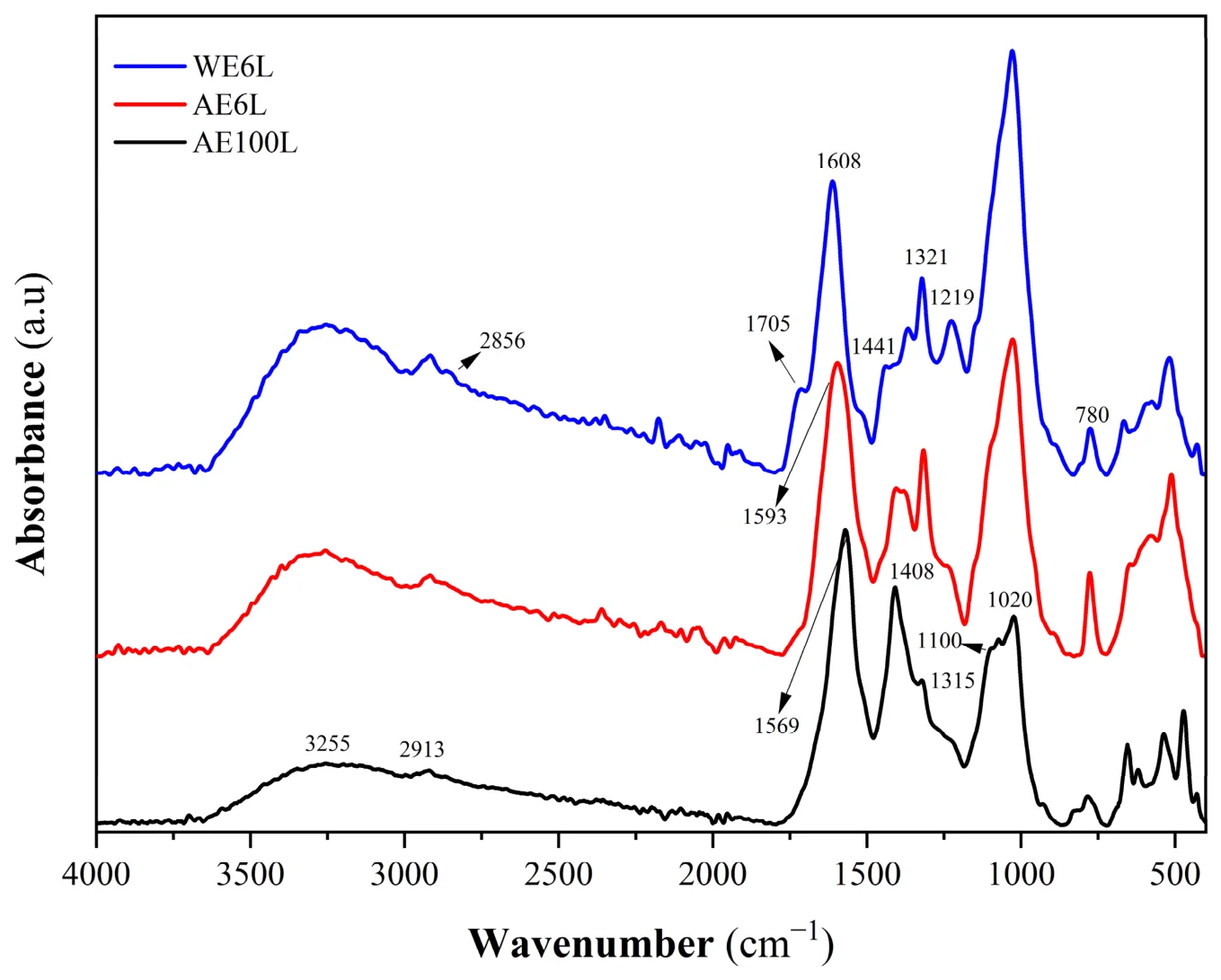
Fourier-transform infrared (FTIR) spectra of the powder extracts. AE100L: powder extract obtained by alkali-assisted extraction in the 100 L reactor; AE6L: powder extract obtained by alkali-assisted extraction in the 6 L reactor; WE6L: powder extract obtained by water-assisted extraction in the 6 L reactor.

Figure 2 shows the main bands of the FTIR-ATR analysis. For reference, the spectra of the extracts obtained from alkaline extraction using the 6 and 100 L reactors and from aqueous extraction using a 6 L reactor are included. To facilitate analysis, the FTIR spectra were normalized to the aromatic ring-stretching band at approximately 1600 cm^−1^ [64]. In this regard, the band at 1569 cm^−1^ observed in the pilot alkaline extract corresponds to the C=C stretching vibration of the phenolic aromatic ring, associated with condensed tannins. A similar band was observed in extracts from the 6 L reactor, although it shifted to higher wavelengths (1593 cm^−1^ for the alkaline extract and 1608 cm^−1^ for the aqueous extract), indicating a higher abundance of resorcinol-type structures characteristic of condensed tannins [64,65]. Another characteristic band of condensed tannins is observed at 1408 cm^−1^ in the pilot and laboratory alkaline extracts, attributed to the bending of the C-H bonds of the CH_2_ groups [33,66], while the band at 1441 cm^−1^ corresponds to the deformation in the plane of C-C, C-H, and O-H bonds. The band observed between 1315 and 1321 cm^−1^ is associated with the deformation of the Ph-CHR-OH group, while the band at 1100 cm^−1^ corresponds to the stretching of the C-O-C bond [33]. On the other hand, a broad band centered at 3255 cm^−1^, associated with the -OH stretching vibrations, was observed in the extracts [67]. In addition, the asymmetric stretching band of CH_2_ at 2913 cm^−1^ is distinguishable, while the symmetric stretching band at 2856 cm^−1^ of this same group is more evident in the aqueous extract. The intense signal recorded at 1020 cm^−1^ corresponds to the C-O, C-C, and C-C-O stretching vibrations of free sugars or sugars associated with eucalyptus gallotannins [33].

On the other hand, for the WE6L extract, the phenol-OH stretching band at 1219 cm^−1^ is observed, in addition to the C=O stretching at 1705 cm^−1^. The presence of both bands, together with the band associated with the symmetric stretching of aromatic ethers at 780 cm^−1^, suggests a high presence of hydrolyzable tannins [68]. Alkaline extraction yielded extracts enriched in condensed tannins and with a lower relative content of sugars, as evidenced by the reduction in the intensities of bands at 1705, 1220, and 1020 cm^−1^, which are characteristic of hydrolyzable tannins and carbohydrate structures. In contrast, an increase in the bands around 1400 and 1100 cm^−1^, together with a noticeable shift in the aromatic band near 1600 cm^−1^, supports a higher contribution of condensed tannins in the alkaline extract. Additionally, the reduced intensities of the bands at 2920, 2850, and 1440 cm^−1^, associated with CH2 stretching and bending vibrations, suggest the presence of lower-molecular-weight compounds, likely resulting from alkaline hydrolysis.

### 3.4. TPC and Antioxidant Capacity

Table 3 summarizes the TPC values and antioxidant capacity of the extracts obtained. The eucalyptus bark extracts showed lower TPC values than those reported by Vásquez et al. [48] for *E. globulus* bark extracted with water and a NaOH solution. These authors used a NaOH concentration of 3% *w*/*w*, significantly higher than the value used in our research (~0.05% *w*/*w* NaOH solution). As with the yield results, alkaline extraction increased TPC. A decrease in the TPC value has been reported with highly alkaline solutions (2.5 and 10% *w*/*v*) compared to aqueous extraction, which increases the extraction yield at the expense of increased extraction of non-tannin compounds, thereby reducing the quality of the extract [38]. In our investigation, no significant difference in TPC values was observed between alkaline and aqueous extractions, likely due to the low NaOH concentration used. The TPC value was maintained, while the extraction yield was higher, indicating that the used alkali concentration favors both yield and the extraction of phenolic compounds. No statistically significant differences were observed between the alkaline and aqueous extracts, and scaling did not affect the TPC. In this regard, the Shapiro–Wilk test did not indicate significant deviations from normality for the evaluated datasets, with *p*-values greater than 0.05 in all cases where the test was applicable. Therefore, the use of one-way ANOVA followed by Tukey’s post hoc test was considered appropriate.

Scale-up did not significantly affect TPC or ABTS-based antioxidant capacity, as no statistical differences were observed between AE100L and AE6L for these parameters. However, significantly lower DPPH and FRAP values were observed in the pilot-scale extracts. This behavior suggests that the antioxidant response depends not only on the total phenolic content but also on the chemical structure and accessibility of the phenolic compounds in the extract. The DPPH assay relies on the ability of antioxidant molecules to neutralize the DPPH radical via hydrogen atom or electron transfer [69]. This response can be strongly influenced by the steric accessibility of the DPPH radical site and by structural features of phenolic compounds, including the availability and position of phenolic hydroxyl groups. Consequently, larger, more condensed or glycosylated phenolic structures may exhibit reduced apparent DPPH-scavenging capacity due to limited accessibility of their reactive sites to the radical. In contrast, the FRAP assay is based on the reduction of the Fe^3+^-TPTZ complex to Fe^2+^-TPTZ under acidic conditions and is mainly governed by the redox potential of the compounds [70]. Therefore, it is less dependent on radical accessibility. Thus, the lower DPPH value and more moderate decrease in FRAP observed for AE100L may be due to differences in the structural characteristics and accessibility of the phenolic compounds in the extracts, rather than to a reduction in their total amount. The FRAP values obtained in this study range from 203 to 310 mg GAE/100 g db, which are up to five times higher than those reported by other researchers for aqueous extraction and approximately half of those reported for methanolic and supercritical carbon dioxide extractions [71].

### 3.5. Thermal Stability

#### 3.5.1. TGA

**Figure 3:**
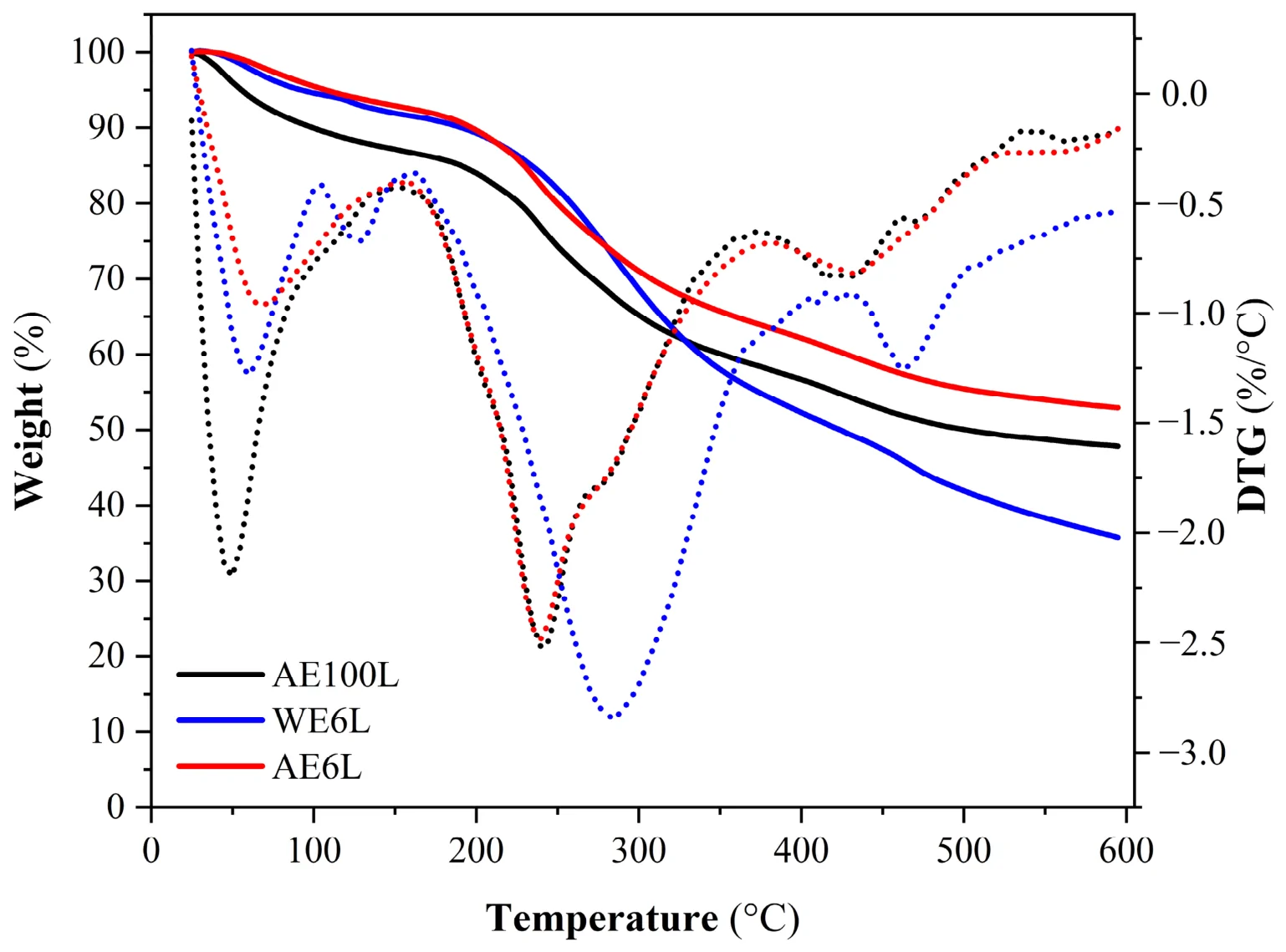
Thermogravimetric analysis (TGA, solid lines) and derivative thermogravimetry (DTG, dashed lines) curves of dried powder extracts. AE100L: powder extract obtained by alkali-assisted extraction in the 100 L reactor; AE6L: powder extract obtained by alkali-assisted extraction in the 6 L reactor; WE6L: powder extract obtained by water-assisted extraction in the 6 L reactor.

Figure 3 shows the thermograms of the pilot- and laboratory-level alkaline extracts obtained with water as the solvent, which are included as references (solid lines). The first derivatives for each sample are also included (DTG, dotted lines).

In industrial applications, thermal stability is an essential property of phenolic compounds, as high temperatures can alter the chemical properties of extracts rich in them [72]. Both alkaline extracts exhibited similar mass-loss profiles, whereas the aqueous extract showed lower thermal stability. In the temperature range of 100–200 °C, all extracts showed limited mass loss, mainly due to the release of physically adsorbed water and low-molecular-weight volatiles [73,74,75]. In this regard, the pilot-scale alkaline extract exhibited the earliest onset of mass loss, primarily due to a higher adsorbed moisture content. The extracts demonstrated adequate stability within this operational range, with less than 10% mass loss up to 200 °C. The onset of significant degradation above 200 °C provides an adequate safety margin for industrial processing.

The maximum rate of thermal degradation occurred between 220 and 300 °C, with mass losses of 15–25%, indicating the onset of decomposition of phenolic and lignocellulosic structures [75,76,77]. At the end of the analysis, the alkaline extracts exhibited a total mass loss of approximately 50%, substantially lower than the ~80% observed for the aqueous extract.

The DTG curves clearly show three central weight-loss regions: (I) 25–150 °C; (II) 150–400 °C; and (III) 400–600 °C, the latter observed only in the sample obtained by water extraction. The weight loss observed in Region I, with a minimum around 50–60 °C, corresponds to the evaporation of water adsorbed in the samples [78]. In the DTG curves, the minimum weight loss recorded in Region II occurred between 240 and 300 °C, which has been associated with the degradation of tannins and polysaccharides [73,74,76,77]. In this regard, it has been reported that pine bark tannin samples registered a signal with a minimum at 280 °C, which was attributed to the degradation of polysaccharides [75].

As mentioned above, the sample obtained by water extraction exhibits lower thermal stability. Gaugler and Grigsby [75] proposed that the low thermal stability of a quebracho tannin sample was explained by the low number of hydroxyls and their substitution pattern in the A-ring [79].

Concerning decomposition region III, observed only in the sample obtained from aqueous extraction, it has been indicated that complex thermal degradation processes of tannins exist, which can continue degradation up to 460 °C [80].

#### 3.5.2. DSC

The different powder extracts were analyzed using Differential Scanning Calorimetry (DSC). This technique measures temperature-dependent thermal effects related to chemical reactions and phase transitions. It is the most widely used method for establishing the glass transition temperature (Tg) of a natural or synthetic polymer [76]. This methodology records the difference in heat flow to the sample and to a reference at the same temperature as a function of temperature [81].

**Figure 4:**
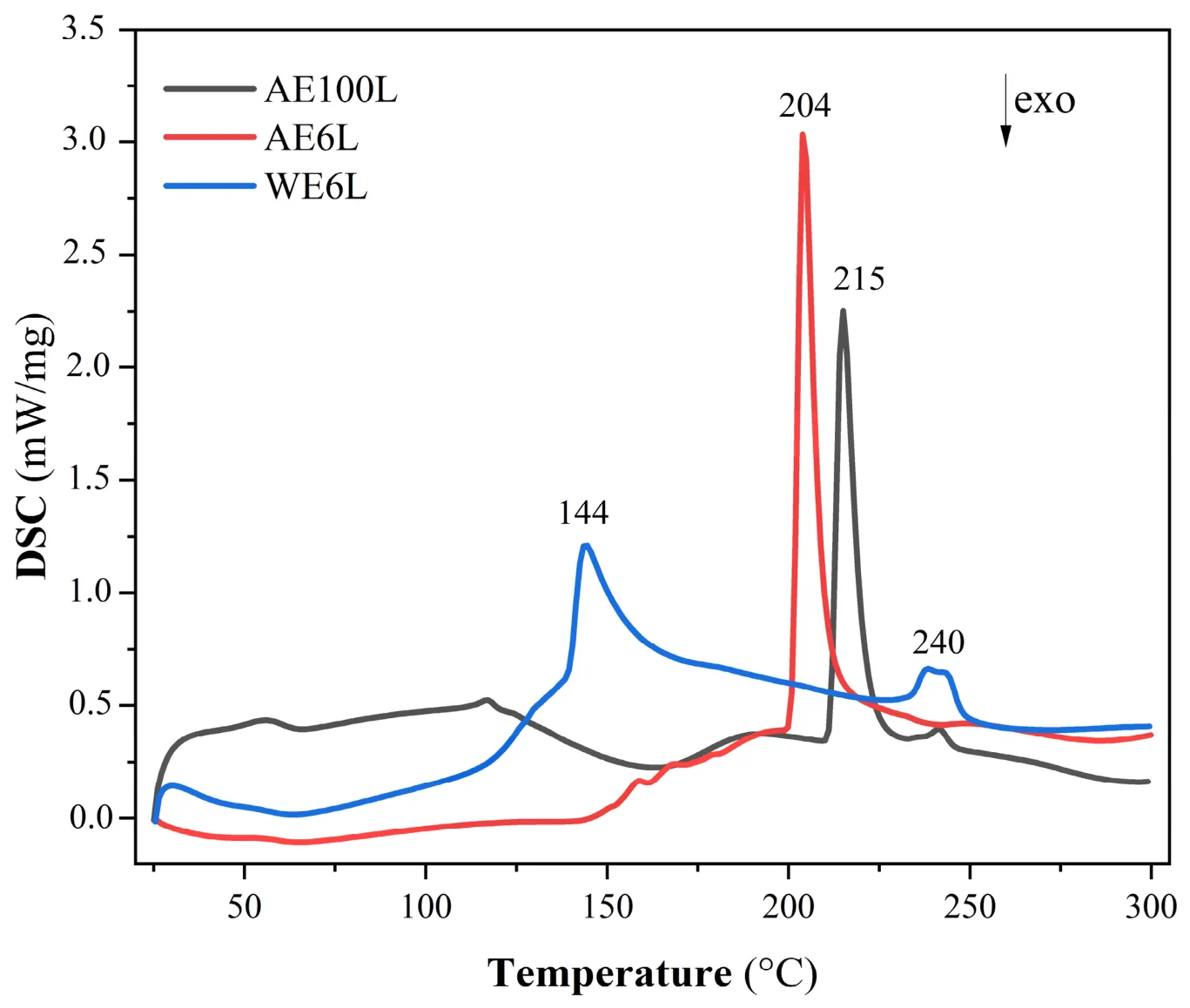
Differential Scanning Calorimetry (DSC) thermograms for powder extracts. AE100L: powder extract obtained by alkali-assisted extraction in the 100 L reactor; AE6L: powder extract obtained by alkali-assisted extraction in the 6 L reactor; WE6L: powder extract obtained by water-assisted extraction in the 6 L reactor.

The DSC thermograms of the powdered extracts obtained through alkaline extraction at pilot (AE100L) and laboratory (AE6L) scales, as well as the aqueous extract (WE6L) used as a reference, are shown in Figure 4. In general, all samples exhibited endothermic peaks characteristic of thermal elimination processes [77]. The broad endothermic band observed up to 150 °C is mainly attributed to the loss of adsorbed water [75,77]. Similar results were reported by Mendoza-Wilson et al. [82] for catechin, a monomeric flavonoid; endothermic maxima were identified at 100 °C and 150 °C corresponding to the loss of adsorbed water. In particular, the aqueous extract (WE6L) shows a well-defined maximum at 144 °C, indicating a higher amount of retained water.

Above 150 °C, endothermic signals at 204 °C, 215 °C, and 240 °C are associated with the thermal decomposition of tannins and polysaccharides in the extracts, leading to the formation of volatile compounds [83]. This behavior is consistent with the TGA results, which show that degradation of these components begins around 200 °C and reaches a minimum in the DTG curve near 240 °C for the alkaline extracts. According to Pizzi [84], condensed tannins exhibit endothermic transitions within this temperature range due to the cleavage of interflavonoid bonds and the decomposition of phenolic structures, which explains the well-defined peaks observed at 204 °C and 215 °C in alkaline extracts. The broad endothermic band in the aqueous extract WE6L suggests that water loss and degradation of tannins and polysaccharides co-occur in this sample.

Extraction with water or an alkaline solution clearly affects the thermal stability of the extracts. The profiles for AE6L and AE100L show a shift in the endothermic signals to higher temperatures compared to WE6L, indicating greater thermal stability of the alkaline extracts. When comparing the alkaline extracts produced at laboratory and pilot scales, no significant differences were observed in the main endothermic peaks (204 °C and 215 °C), except for a broad signal below 150 °C in the pilot-scale extract (AE100L), suggesting a slightly higher moisture content in this sample.

### 3.6. Retention Analysis

Absorption (A) refers to the amount of extract that penetrates the empty spaces in the wood structure. In contrast, retention refers to the amount of active components in the solution retained in the wood after impregnation [85]. Table 4 reports the absorption, retention, the percentage of the retained extract in the wood, and the weight loss after lixiviation of samples impregnated with the different extracts. The extracts’ absorption was similar across extraction methods, with no significant differences. Given that the AE100L and WE6L samples contained the same extract concentration (2.5% *w*/*v*) and were dissolved in an aqueous medium, they are expected to exhibit comparable densities and viscosities and, consequently, to show similar absorption values. Because the solid content is relatively low, the extract’s viscosity is equivalent to that of water, so diffusion would not be affected. Therefore, absorption values can be up to three times higher than those reported in other studies. In this regard, Owoyemi et al. [85] reported absorption values ranging from 15 to 28% for different preservatives and wood species, which contrasts with the 63% (calculated according to the methodology of Owoyemi et al.) obtained in this research. It is known that high viscosities can generate resistance to liquid penetration into the wood structure [85,86]. Regarding retention, since the concentration and absorption values of both extracts are the same, no changes in retention were observed. The retention values obtained (20.5–21.6 kg/m^3^) are within the range reported for bio-based wood preservatives [87,88]. For comparison, commercial tannin-based preservatives typically achieve retentions of 15–50 kg/m^3^ depending on concentration and wood species. The NaOH solution used as a reference showed a very low retention value (0.36 kg/m^3^), compared with the samples treated with the extract. Hence, the contribution of NaOH is not significant (close to 2%), as expected, given that the NaOH solution had a nominal concentration of only 0.05% *w*/*v*. The percentage of retained extract in the wood (%REW) were 2.44% for AE100L and 2.54% for WE6L, which is consistent with the nominal concentration of the extract solutions used for impregnation. This confirms that both extract-treated wood samples received a comparable initial solid loading during the Bethel process.

Lixiviation in treated wood is the process of chemically leaching preservatives (i.e., metals, natural extracts) from the wood. The weight loss after leaching (WLL) values reported in Table 4 showed that wood samples treated with the aqueous extract (W6L) exhibited a significantly higher WLL (3.94%) compared to those impregnated with the alkaline extract (AE100L, 2.15%), indicating a greater loss of extractable material during the leaching process. This behavior can be directly related to the higher content of water-soluble phenolic compounds in the W6L extract, which are more readily dissolved and migrate in aqueous environments. In contrast, the lower WLL observed for the AE100L-treated samples reflects more stable fixation of phenolic compounds within the wood structure, likely due to the formation of higher-molecular-weight, less-soluble phenolic complexes during alkaline extraction, as will be discussed below. The markedly lower WLL recorded for the NaOH-treated samples (0.71%) further supports this interpretation, as the absence of extractable phenolic compounds limits mass loss to minor dissolution of wood components. Overall, the WLL results confirm that alkaline extraction enhances the leaching resistance of the impregnated wood, improving durability and reducing loss of functional components.

**Figure 5:**
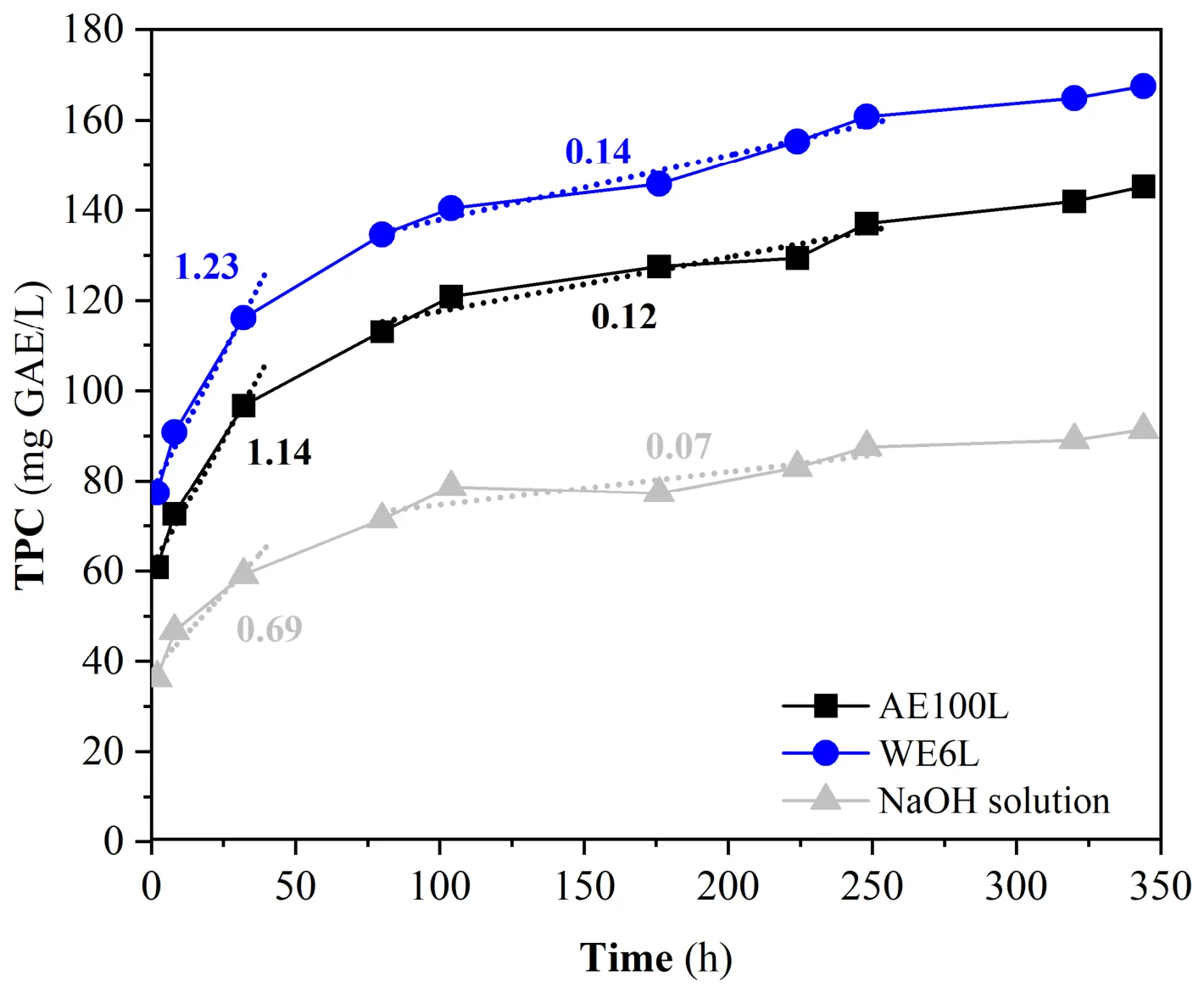
Total Phenolic Content (TPC) in leachate over time. AE100L: Sample treated with the extract obtained by alkali-assisted extraction in the 100 L reactor (solution of 2.5% *w*/*w*); WE6L: Sample treated with the extract obtained by water-assisted extraction in the 6 L reactor (solution of 2.5% *w*/*w*); NaOH solution: Sample treated with a solution of 0.05% *w*/*v* NaOH. Dotted lines represent the TPC release rate expressed in mg GAE/(L*h).

The TPC present in the leachate from the samples impregnated with the alkaline extract (AE100L) and the aqueous extract (WE6L) is shown in Figure 5. For reference, the TPC in the leachate from samples impregnated with a NaOH solution is also included. The aqueous extract is more leachable than the alkaline extract, registering a cumulative TPC value of 168 mg GAE/L at the end of the test, compared to 140 mg GAE/L for the alkaline extract. This trend is to be expected, given that the W6L extract was obtained by 100% aqueous extraction, in addition to its hygroscopic nature and the poor fixation of tannins in the wood structure [89,90,91].

To further quantify the release behavior, the cumulative TPC leaching curves were analyzed by calculating the apparent linear release rates (dotted lines) during two representative stages: an initial stage from 2 to 32 h and a later stage from 80 to 248 h.

During the initial stage, the apparent TPC release rates were 1.14, 1.23, and 0.69 mg GAE/(L*h) for AE100L, WE6L, and the NaOH solution, respectively. In the later stage, these values decreased markedly to 0.12, 0.14, and 0.07 mg GAE/(L*h), respectively. This reduction by approximately one order of magnitude confirms that TPC leaching mainly occurred during the first hours of exposure, followed by a much slower release stage.

The WE6L treatment exhibited slightly higher release rates than the AE100L treatment in both time intervals, consistent with its higher final cumulative TPC release. These results suggest that the aqueous extract contained a higher proportion of water-soluble, weakly retained phenolic compounds. In contrast, the lower release rate and cumulative TPC observed for AE100L indicate reduced mobility of phenolic compounds within the wood matrix, likely due to stronger interactions between the phenolic compounds extracted by an alkaline method and the lignocellulosic components.

The higher content of leached polyphenols in the wood samples treated with the W6L extract suggests that this aqueous extract contains a higher proportion of water-soluble phenolic compounds, such as hydrolyzable tannins, flavonoids, and phenolic acids, which are released into an aqueous medium [59,92]. In contrast, the AE100L extract, obtained by alkaline extraction, has a lower TPC in the leachate, despite originally containing a higher TPC than the samples treated with the W6L extract. This could be explained by the alkaline extraction medium, which favors partial lignin depolymerization and the formation of more stable, less soluble phenolic complexes, such as condensation products or modified lignin [93,94]. These compounds have higher molecular weights and tend to be more strongly retained within wood cell walls, thereby reducing their migration into the leachate [95]. In addition, the slightly alkaline pH of the extract (~8) may further promote interactions between polyphenolic compounds and wood cell wall components, particularly cellulose [96]. Additionally, alkaline conditions can alter the stability and reactivity of phenolic hydroxyl groups, thereby changing their interactions with structural polymers [97].

The difference between W6L and AE100L also reflects the influence of the extraction medium on the chemical composition and mobility of the recovered compounds. As discussed above, alkali-assisted extraction increased the extraction yield compared with water extraction (see Table 1), and the extraction solution can influence the types and amounts of compounds recovered from eucalyptus bark. In this sense, Sartori et al. [98] provide additional context, noting that water extraction of Eucalyptus bark yielded a Stiasny index of 20%, while sodium sulfite extraction produced a 45% index, indicating that the extraction medium can affect tannin recovery and reactivity. In the present study, the lower TPC release from AE100L-treated wood indicates reduced mobility and/or greater retention of alkaline-extracted compounds within the wood structure compared with that from WE6L. However, this result should be interpreted with caution, since the leaching test does not allow us to distinguish whether the extractives were retained through interactions with lignocellulosic components, physical accumulation within the cell lumina, or a combination of both mechanisms. Therefore, further studies using microscopic and spectroscopic techniques are required to determine the distribution and retention mechanism of the extractives within the treated wood.

### 3.7. Surface Color Analysis

**Figure 6:**
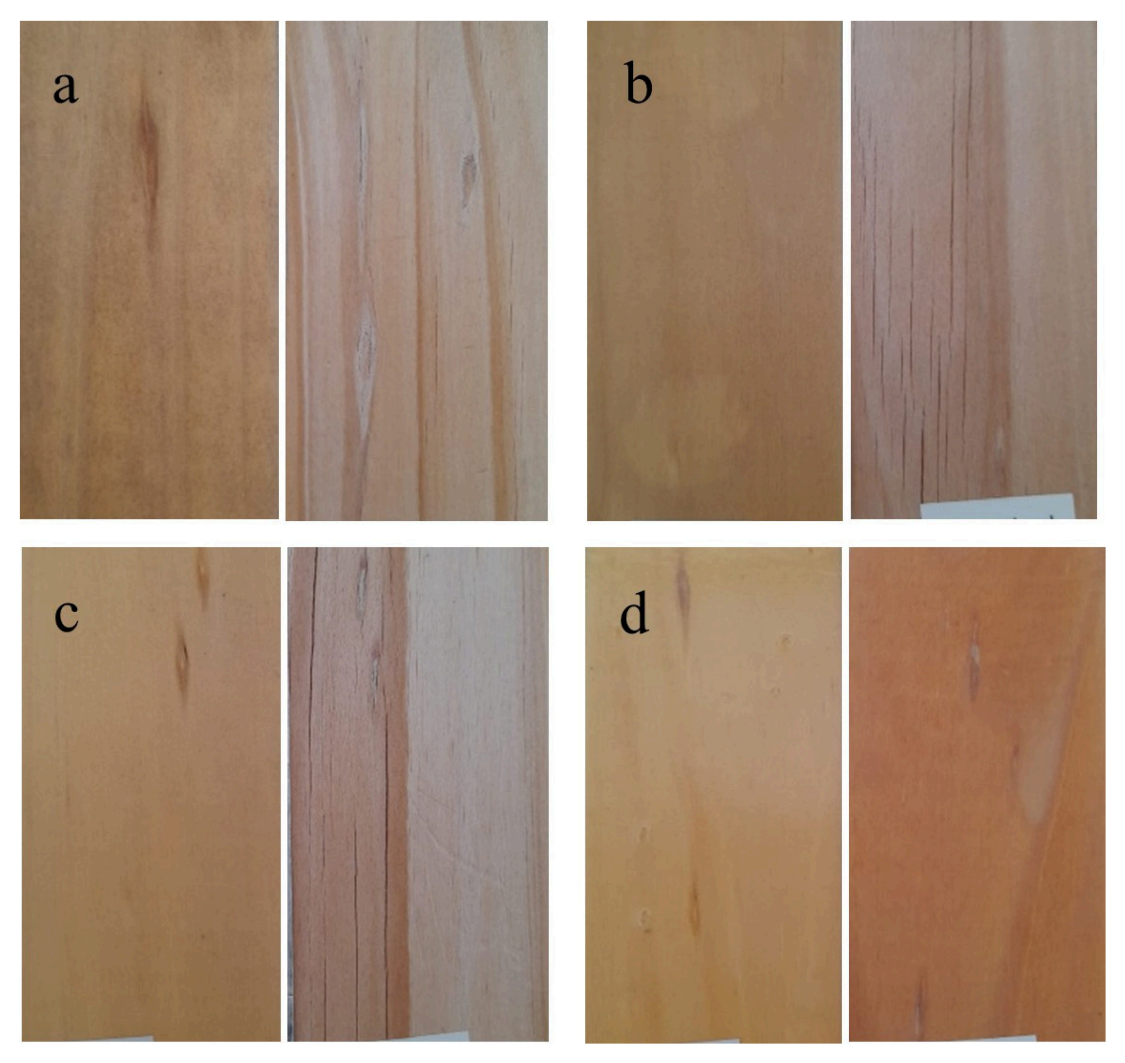
Appearance of samples before (image with lowercase letter) and after aging in the UV chamber for 1000 h. (**a**) AE100L, (**b**) NaOH solution, (**c**) Water, (**d**) CP. AE100L: Sample treated with the extract obtained by alkali-assisted extraction in the 100 L reactor (solution of 2.5% *w*/*w*); NaOH solution: Sample treated with a solution of 0.05% *w*/*v* NaOH; Water: Sample treated with water; CP: Commercial product.

The evaluation of color stability under accelerated weathering provides critical information on the photoprotective capacity of wood treatments. Figure 6 shows the appearance of the sample before (12 h control) and after the test (sample next to the numbered sample) in the UV chamber. The impregnated samples (Figure 6a,b) are light brown and tend to lighten upon exposure. This trend was also observed in the sample impregnated with water alone (Figure 6c). In contrast, the sample painted with the CP (Figure 6d) changes from light yellow to light brown with aging, i.e., it darkens with the test. In this regard, it is important to note that the commercial product not only penetrates the wood but also forms a protective layer.

**Figure 7:**
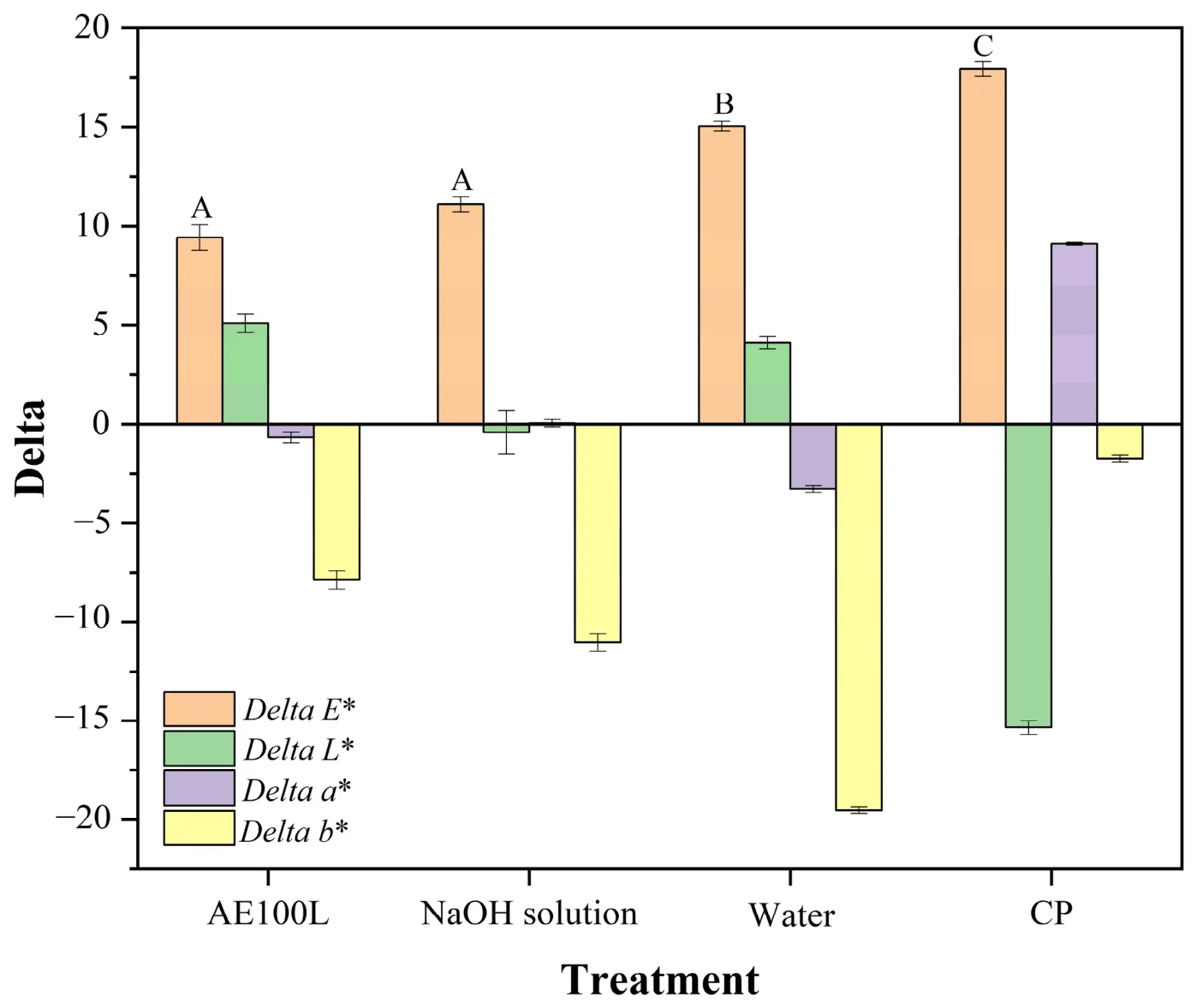
Changes in the color coordinates of the samples after 1000 h in the UV chamber. AE100L: Sample treated with the extract obtained by alkali-assisted extraction in the 100 L reactor (solution of 2.5% *w*/*w*); NaOH solution: Sample treated with a solution of 0.05% *w*/*v* NaOH; Water: Sample treated with water; CP: Commercial product. Different superscript letters above the Delta E* column indicate statistically significant differences among treatments (*p* < 0.05), while identical letters indicate no significant differences.

The results of Delta E*, Delta L*, Delta a*, and Delta b* for the evaluated samples are shown in Figure 7. In the control sample (water), it is noteworthy that, despite not receiving any treatment, the Delta E* value did not change significantly. It has been reported that lignin is the first component of wood to degrade, owing to its strong UV absorbance [99]. The products of this degradation (ketones, aldehydes, and quinones) are washed away during the condensation cycle and removed from the test piece’s surface, exposing the cellulose, which is more stable in color under UV light [100]. Although the untreated wood sample did not have the highest Delta E value, it showed the most significant surface damage, evidenced by well-marked cracks.

Although many studies report an initial darkening of pine wood exposed to UV radiation due to lignin photodegradation, recent comparative research has shown that, after prolonged exposure, the rapid color change observed at shorter times tends to decrease, and luminosity stabilizes or even increases, indicating a surface lightening phase [101]. Additionally, under moisture-cycling weather conditions, degradation products can leach from the wood surface, further increasing lightness relative to intermediate stages of aging.

The letters above the Delta E* column in Figure 7 indicate the homogeneous groups defined by Tukey’s test; treatments sharing the same letter do not differ significantly from each other (*p* > 0.05), while those with different letters belong to statistically different groups (*p* < 0.05). No significant differences in Delta E were observed between the wood samples treated with eucalyptus bark extract and the alkaline solution, whereas differences were observed for the other treatments.

To observe cracks in greater detail, photographs of the samples were taken after 1000 h. The results indicate that, after 250 h of exposure, cracks appeared in all wood samples. The first wood sample to develop cracks was the one impregnated with the NaOH solution, which showed them after 150 h of exposure. The wood sample impregnated with eucalyptus bark extract stood out for its lower overall color change and resistance to cracking. Regarding the commercial control, although it showed a significant color change, the wood remained protected from surface damage, and no cracks were observed.

The UV-protective effect of the eucalyptus bark extract is attributed to a dual mechanism mediated by its polyphenolic constituents. First, phenolic compounds containing conjugated aromatic rings and carbonyl groups act as UV absorbers, dissipating radiation energy as heat before it can initiate photochemical reactions in lignin. Second, the high antioxidant capacity of the extract (as demonstrated by DPPH, ABTS, and FRAP assays) enables scavenging of free radicals generated during photooxidation, thereby interrupting the chain reactions responsible for lignin degradation and chromophore formation [102].

Total color change is the most important parameter for evaluating the color stability of aged samples. Figure 7 shows that the trend in Delta E variation was consistent with that of Delta L*, except for the sample treated with the commercial product, whose trend was consistent with Delta a*. This result would indicate that the increase in lightness was the dominant factor influencing the discoloration of the impregnated samples. Since the samples are impregnated (no film formation), the extracts are directly exposed to radiation, leading to rapid, superficial degradation and a lighter surface over time. However, the results indicate that eucalyptus bark extract exhibits good UV protection.

**Figure 8:**
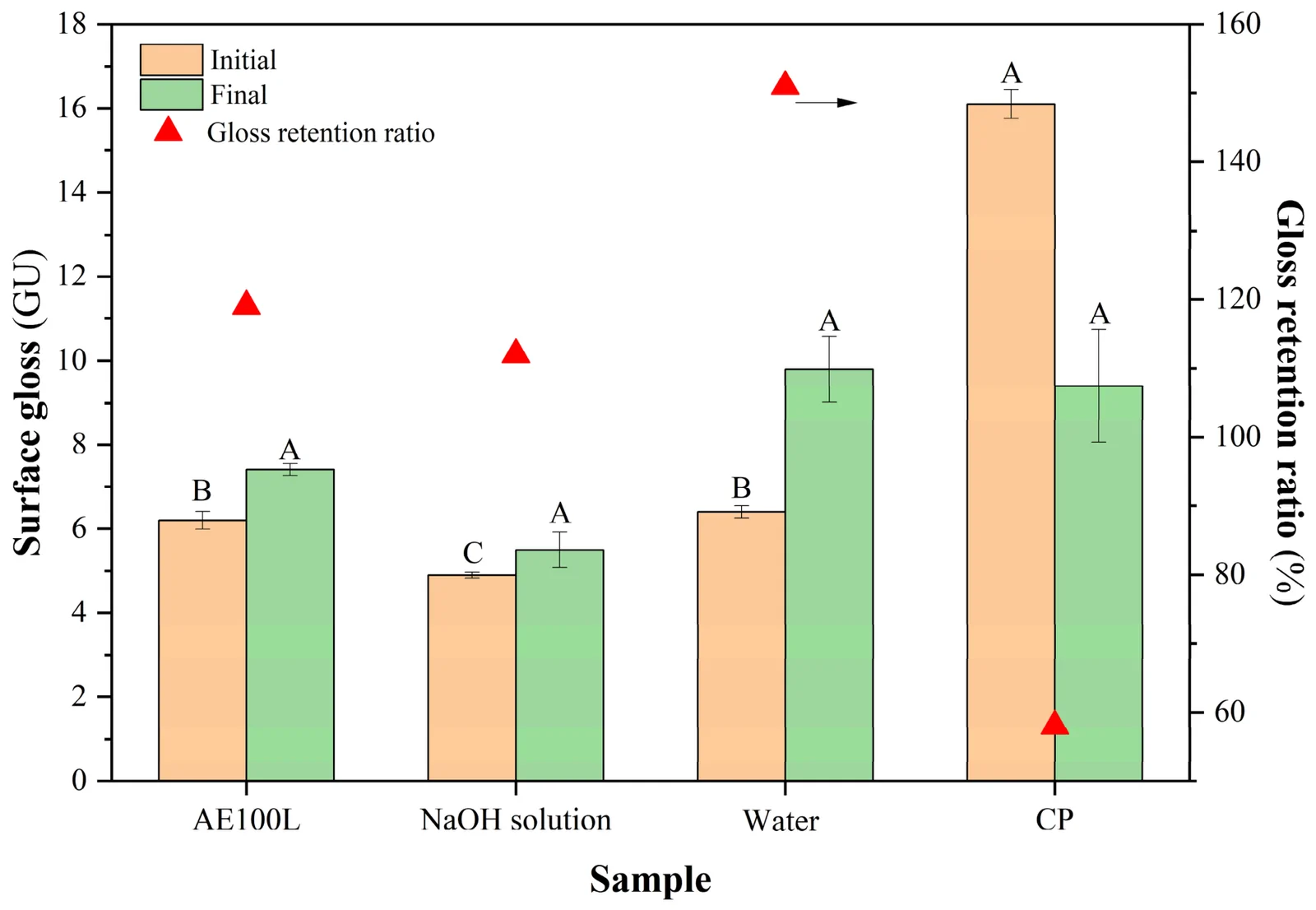
Surface gloss values (initial and final) and Gloss retention ratio of samples. AE100L: Sample treated with the extract obtained by alkali-assisted extraction in the 100 L reactor (solution of 2.5% *w*/*w*); NaOH solution: Sample treated with a solution of 0.05% *w*/*v* NaOH; Water: Sample treated with water; CP: Commercial product. Different superscript letters indicate statistically significant differences among treatments (*p* < 0.05), while identical letters indicate no significant differences.

Figure 8 shows the gloss analysis of the test specimens before and after artificial weathering. Wood samples showed lower initial gloss values, except for the sample treated with the commercial product, which had a higher initial gloss. At the end of the test, the final gloss increased after 1000 h in the UV chamber, and gloss retention ratios higher than 100% were obtained, except for the sample treated with the commercial product (58%). The observed result is consistent with the color test, where lightness (L*) was the color coordinate that significantly influenced the discoloration of the wood samples. UV radiation degraded the phenolic compounds on the surface of the wood sample impregnated with the extract, initially giving the sample a light brown color and a matte appearance. Photodegradation and washing by condensation and evaporation inside the chamber can lighten the surface and make it smoother [103], making it appear optically brighter, even without a coating.

In the wood sample treated with the commercial product, a decrease in gloss was observed after 1000 h in the UV chamber. In this case, once applied, the product forms a surface layer with moderate gloss (16 GU), which is up to 3 times higher than that of the other samples evaluated, making it significantly different from them. After aging, the photodegradation of the commercial product’s compounds increased the red index (Delta a*) in the wood sample [104], as shown in Figure 7. Overall, there are significant differences in initial gloss across all samples, but after artificial weathering, no statistically significant differences were observed, with values ranging from 5 to 10 GU.

**Figure 9:**
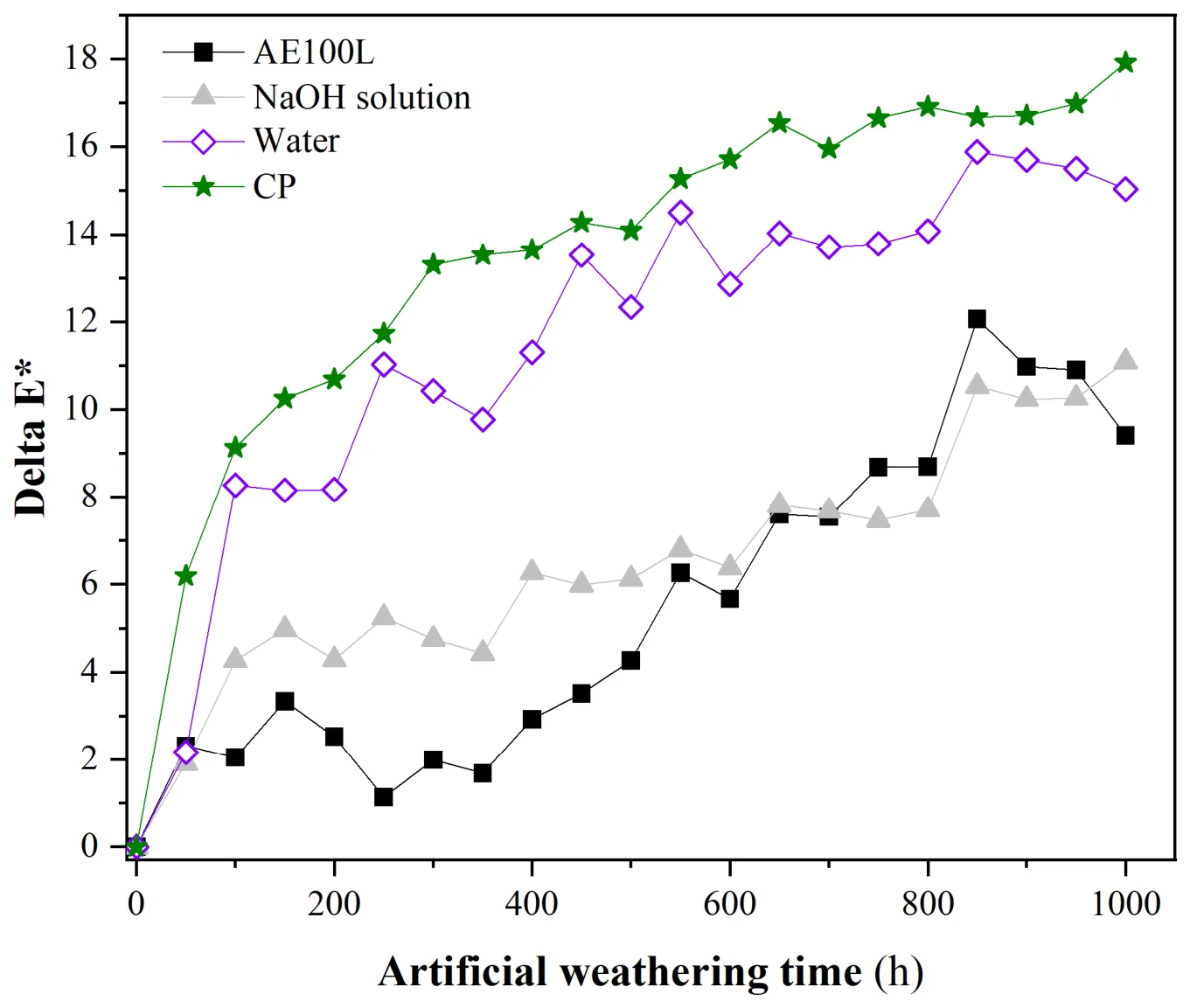
Changes in the Delta E* of wood samples at different artificial weathering times in the UV chamber. AE100L: Sample treated with the extract obtained by alkali-assisted extraction in the 100 L reactor (solution of 2.5% *w*/*w*); NaOH solution: Sample treated with a solution of 0.05% *w*/*v* NaOH; Water: Sample treated with water; CP: Commercial product.

Figure 9 shows the Delta E value as a function of aging time. In the untreated sample and the sample treated with the CP, the Delta E value changes rapidly up to 400 h, whereas in the other samples, the color change is less drastic, with the sample impregnated with eucalyptus bark extract (AE100L) standing out. Although the sample treated with the NaOH solution shows a less pronounced change in the Delta E slope, this does not indicate better protection, as it was the first to show cracks. Similarly, the sample treated with the commercial product recorded the highest Delta E* value while maintaining protection for up to 250 h. For this sample, the trend in the Delta E value corresponds to Delta a*, i.e., a reddening of the surface as the aging time increases [23]. For the remaining samples, as discussed above, the change in Delta E may be related to brightness, highlighting the smaller color change in the sample treated with the alkaline extract and its resistance to cracking after 250 h of UV chamber exposure.

### 3.8. Antifungal Potential

#### 3.8.1. Inhibition of Staining Fungi

The effects of mold, staining, and rotting fungi were evaluated in wood samples treated with the alkaline extract and compared with reference samples treated with water (negative control), alkalis (as an additional solvent), and commercial product (Nipacide P511). Table 5 and Table 6 report the percentage of growth inhibition of the staining fungi *D. seriata* and the mold *T. viride*, respectively. The alkaline extract of eucalyptus bark at 2.5% *w*/*v* exhibited remarkable antifungal efficacy, achieving 90.91% inhibition against *D. seriata* and complete inhibition (100%) against *T. viride*. Interestingly, for *T. viride*, the AE100L extract showed the same level of inhibition as the commercial product Nipacide P511 (100%). The water and NaOH controls indicate that the observed antifungal activity was attributable to the bioactive compounds extracted from eucalyptus bark, rather than to the alkaline extraction medium itself. It should be noted that the test fungi belong to the same genera as those specified in ASTM D4445-10 but are not the exact species listed therein; for coniferous timber, the standard specifies *Diplodia natalensis*, whereas *D. seriata* was used here. Accordingly, a slightly different staining profile and slower colonization may be expected. Consistent with this, the *D. seriata* negative controls received a mean rating of 3.67 ± 0.58 rather than the maximum of 5, indicating substantial but incomplete colonization over the four-week incubation period, whereas the fast-colonizing mold *T. viride* reached full development (5.00 ± 0.00). A longer incubation would likely have driven the *D. seriata* controls closer to full staining.

It has been reported that *E. globulus* bark is mainly composed of hydrolyzable gallotannins with a high concentration of quercetin and isorhamnetin components when an alkaline extraction is used [38,92]. Quercetin and isorhamnetin exhibit significant antifungal activity against multiple fungal species, with strong evidence of their ability to inhibit fungal growth [34,35,105,106]. Given that the AE100L extract was obtained by alkaline extraction, the excellent antifungal activity observed in samples treated with this extract would be related to the presence of chemical compounds similar to those reported by Vásquez et al. [38]. Although a minor inhibitory contribution of the alkaline (NaOH) medium cannot be entirely ruled out, any such effect was minor and statistically indistinguishable from the water control (same superscript). This is further supported by the decay test (Table 7), in which the NaOH control exhibited extensive weight loss, statistically equivalent to that of the water control, confirming that the alkaline medium did not confer meaningful antifungal protection.

#### 3.8.2. Brown-Rot Decay Resistance

The percentage weight loss (%WSL) after the brown-rot fungus decay test of wood samples impregnated with the AE100L extract is shown in Table 7, along with the NaOH solution and an untreated sample as references. After 12 weeks of incubation with *P. placenta*, AE100L-treated wood showed no measurable decay-related weight loss, with a WLD value close to zero within experimental variability. In contrast, the NaOH- and water-treated reference samples were completely colonized by *P. placenta* mycelium and exhibited substantial weight losses of 46–53%. These results indicate that the AE100L treatment provided strong resistance to brown-rot decay under the laboratory conditions evaluated. On the other hand, in the reference samples (NaOH solution and water-impregnated wood), *P. placenta* mycelium completely covered the wood samples, resulting in weight losses of 46–53%. As discussed for the fungal stain, the AE100L extract shows excellent antifungal activity, which we suggest may be due to the presence of quercetin and isorhamnetin. Other extracts, such as mimosa bark (*Acacia mollisima*), have been shown to exhibit antifungal properties against the brown-rot fungus *Gloeophyllum trabeum*, resulting in a 0.8% weight loss at a 12% concentration [88]. This result is of interest, since with the AE100L extract developed in our research, a lower concentration (2.5% *w*/*w*) is required to achieve complete antifungal protection.

The results show that the AE100L extract contains chemical compounds, including phenolic compounds with antifungal potential, which could act through mechanisms of cell structure disruption [105], oxidative stress [107] or modulation of vital fungal processes [108], providing a rational basis for their use as active components in bio-based extracts intended to control staining and rot fungi in lignocellulosic materials. Furthermore, the broad-spectrum efficacy observed against both staining and decay fungi suggests that the AE100L extract targets multiple physiological processes, enhancing its potential as a versatile bio-based wood preservative.

The antifungal activity of polyphenolic compounds against wood-decay fungi involves multiple, well-established mechanisms. Based on the expected composition of the AE100L extract, its bioactivity can be explained as follows. First, flavonoids such as quercetin can disrupt fungal cell membranes by interacting with the phospholipid bilayer, increasing permeability and causing leakage of intracellular components, ultimately leading to cell death [109]. Second, polyphenols may induce oxidative stress within fungal cells. Although they act as antioxidants in wood protection, under intracellular conditions, they can behave as pro-oxidants, promoting the formation of reactive oxygen species (ROS) that damage lipids, proteins, and nucleic acids, overwhelming fungal defense systems. Third, inhibition of the Fenton reaction is particularly relevant for brown-rot fungi such as *P. placenta*. Polyphenols, especially tannins and flavonoids, chelate Fe^2+^ ions required for hydroxyl radical generation, thereby blocking the non-enzymatic cellulose depolymerization pathway central to brown-rot decay [110]. Finally, tannins may inhibit fungal extracellular enzymes, including cellulases and laccases, by precipitating proteins, thereby suppressing both enzymatic and non-enzymatic wood degradation processes [111].

It should be noted that this study only evaluated one extract concentration (2.5% *w*/*w*); therefore, it is not possible to establish a dose–response relationship between extract concentration and wood protection performance at this stage. In principle, increasing the extract concentration could enhance the retention of phenolic compounds within the wood matrix, thereby improving antifungal activity and UV protection. However, higher concentrations may also affect impregnation efficiency, penetration, color, leachability, viscosity, and process cost. Future studies should therefore evaluate a broader range of concentrations to determine the optimal balance between extract loading, durability, antifungal performance, UV resistance, and the visual appearance of the treated wood.

## 4. Conclusions

This study demonstrated that pilot-scale alkaline extraction of *E. globulus* bark produces a polyphenol-rich extract with strong multifunctional potential for wood protection. LC-LTQ-Orbitrap-MS analysis showed that the alkaline extract contained low-molecular-weight organic acids, phenolic acids, a hydrolyzable tannin derivative, ellagic acid, methylated flavonol glycosides, and an iridoid non-phenolic metabolite, supporting its chemical complexity and potential functionality as a bio-based additive for wood preservation. The alkaline process doubled the extraction yield compared to aqueous extraction, while preserving the extract’s phenolic content. AE100L also exhibited sufficient thermal stability for industrial processing and reduced leaching from treated wood, suggesting enhanced fixation within the wood matrix. At a concentration of 2.5% *w*/*w*, the extract exhibited high inhibition levels of stain fungi (90–100%) and provided effective protection against the brown-rot fungus *P. placenta*. Furthermore, treated wood exhibited enhanced resistance to UV exposure and surface cracking. Overall, these results indicate that alkaline *E. globulus* bark extracts are promising bio-based additives for wood protection, showing antifungal and UV-protective functionality under the laboratory conditions evaluated. This approach may also contribute to the valorization of an abundant forestry by-product within circular bioeconomy strategies.

## Figures and Tables

**Table 1:** Yield (*Y*%) obtained for the different extractions.

Sample	Dry Feed Mass (g)	Extracted Liquor Mass (g)	Solids in Liquor (% *w*/*w*)	*Y* (%)	Liquor pH
AE100L	2812.5	79,150	0.314 ± 0.008	8.84 ± 0.92	7.5
AE6L	300	5085	0.457 ± 0.031	7.75 ± 0.26	7.9
WE6L	300	4951	0.228 ± 0.024	3.76 ± 0.11	4.8

Values are expressed as mean ± standard deviation. AE100L: extract obtained by alkali-assisted extraction in the 100 L reactor; AE6L: extract obtained by alkali-assisted extraction in the 6 L reactor; WE6L: extract obtained by water-assisted extraction in the 6 L reactor.

**Table 2:** Identification of compounds in the alkaline *E. globulus* bark extract by LC-ESI-LTQ-Orbitrap-MS in negative mode.

Rt (min)	[M-H]^−^ (*m*/*z*)	MS/MS Fragments in ESI (−) Mode *m*/*z*	Tentative Compound	Molecular Formula	References
1.78	133.0141	115.0035, 89.0244 71.0138	Malic acid	C_4_H_6_O_5_	[57]
2.07	191.0195	173.0086, 111.0087, 87.0087, 85.0295	Quinic acid	C_7_H_12_O_6_	[32]
4.45	169.0140	125.0242	Gallic acid	C_7_H_6_O_5_	[32,40,58]
10.40	153.0192	109.0291	Protocatechuic acid	C_7_H_6_O_4_	[56,58]
16.19	935.0791	275.0196, 301.0352, 169.0136, 300.9983	Casuarinin	C_41_H_28_O_26_	[32]
16.58	227.0925	127.0764	Loganetin (iridoid non-phenolic)	C_10_H_14_O_3_	[59]
17.71	477.0672	314.0064, 315.0104, 300.9961	Isorhamnetin-hexoside	C_22_H_22_O_12_	[32,60]
18.06	300.9989	300.9987, 257.0101, 229.0138, 185.0237	Ellagic acid	C_14_H_6_O_8_	[32,61]
18.18	461.0722	315.0145, 299.9911, 300.9954	Isorhamnetin-rhamnoside	C_22_H_22_O_12_	[32,60]

Rt: retention time.

**Table 3:** Total phenolic content (TPC) and antioxidant capacity of eucalyptus bark extracts.

Sample	TPC (mg GAE/100 g db)	Antioxidant Capacity (mg GAE/100 g db)		
		ABTS	DPPH	FRAP
AE100L	970.7 ± 76.3 ^A^	282.1 ± 40.1 ^A^	128.0 ± 9.5 ^B^	203.1 ± 2.9 ^C^
AE6L	1142.2 ± 41.5 ^A^	251.6 ± 5.6 ^A^	283.1 ± 2.7 ^A^	225.5 ± 5.1 ^B^
WE6L	900.3 ± 70.3 ^A^	313.2 ± 3.5 ^A^	252.4 ± 9.8 ^A^	310.0 ± 2.3 ^A^

Values are expressed as mean ± standard deviation. AE100L: extract obtained by alkali-assisted extraction in the 100 L reactor; AE6L: extract obtained by alkali-assisted extraction in the 6 L reactor; WE6L: extract obtained by water-assisted extraction in the 6 L reactor. Different superscript letters within the same column indicate statistically significant differences among treatments (*p* < 0.05), while identical letters indicate no significant differences.

**Table 4:** Absorption (A), retention (R), weight loss after lixiviation (WLL), and percentage of the retained extract in the wood (REW) for samples treated with different extract solutions.

Sample	A (kg/m^3^)	R (kg/m^3^)	REW (%)	WLL (%)
AE100L	841 ± 65 ^A^	20.5 ± 1.6 ^A^	2.44	2.15 ± 0.26 ^A^
WE6L	851 ± 83 ^A^	21.6 ± 2.1 ^A^	2.54	3.94 ± 0.44 ^B^
NaOH solution	805 ± 71 ^A^	0.36 ± 0.09 ^B^	0.04	0.71 ± 0.09 ^C^

Values are expressed as mean ± standard deviation. AE100L: sample treated with the extract obtained by alkali-assisted extraction in the 100 L reactor (solution of 2.5% *w*/*w*); WE6L: sample treated with the extract obtained by water-assisted extraction in the 6 L reactor (solution of 2.5% *w*/*w*); NaOH solution: Sample treated with a solution of 0.05% *w*/*v* NaOH. Different superscript letters within the same column indicate statistically significant differences among treatments (*p* < 0.05), while identical letters indicate no significant differences.

**Table 5:** Inhibition percentage of the growth of the staining fungi *D. seriata*.

Treatment	Value 1	Value 2	Value 3	Mean ± SD	Inhibition (%)	Final Condition
AE100L	1	0	0	0.33 ± 0.58 ^A^	90.91	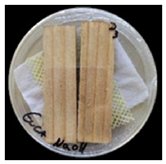
NaOH solution	4	3	4	3.67 ± 0.58 ^B^	0	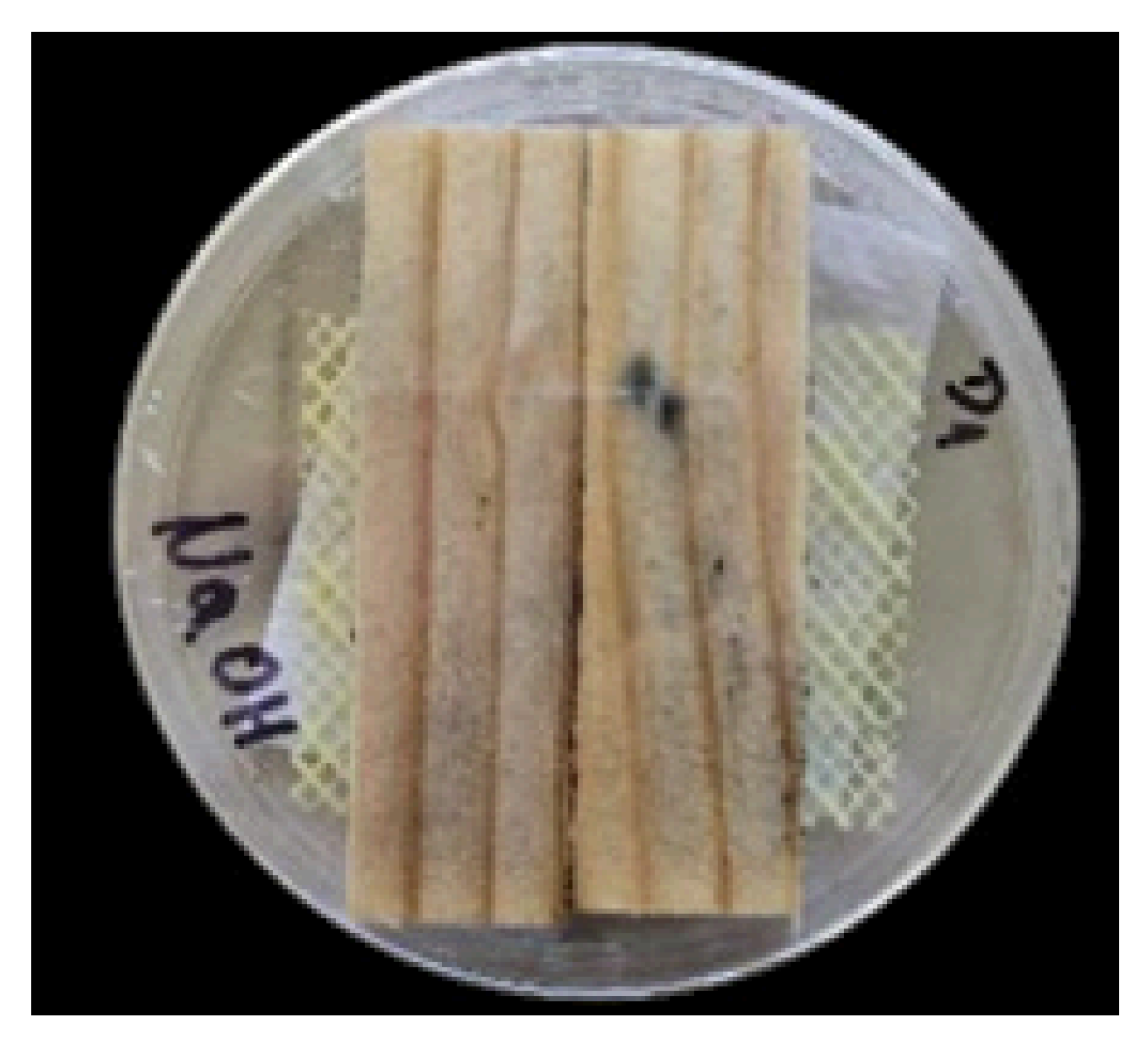
Water	4	4	3	3.67 ± 0.58 ^B^	0	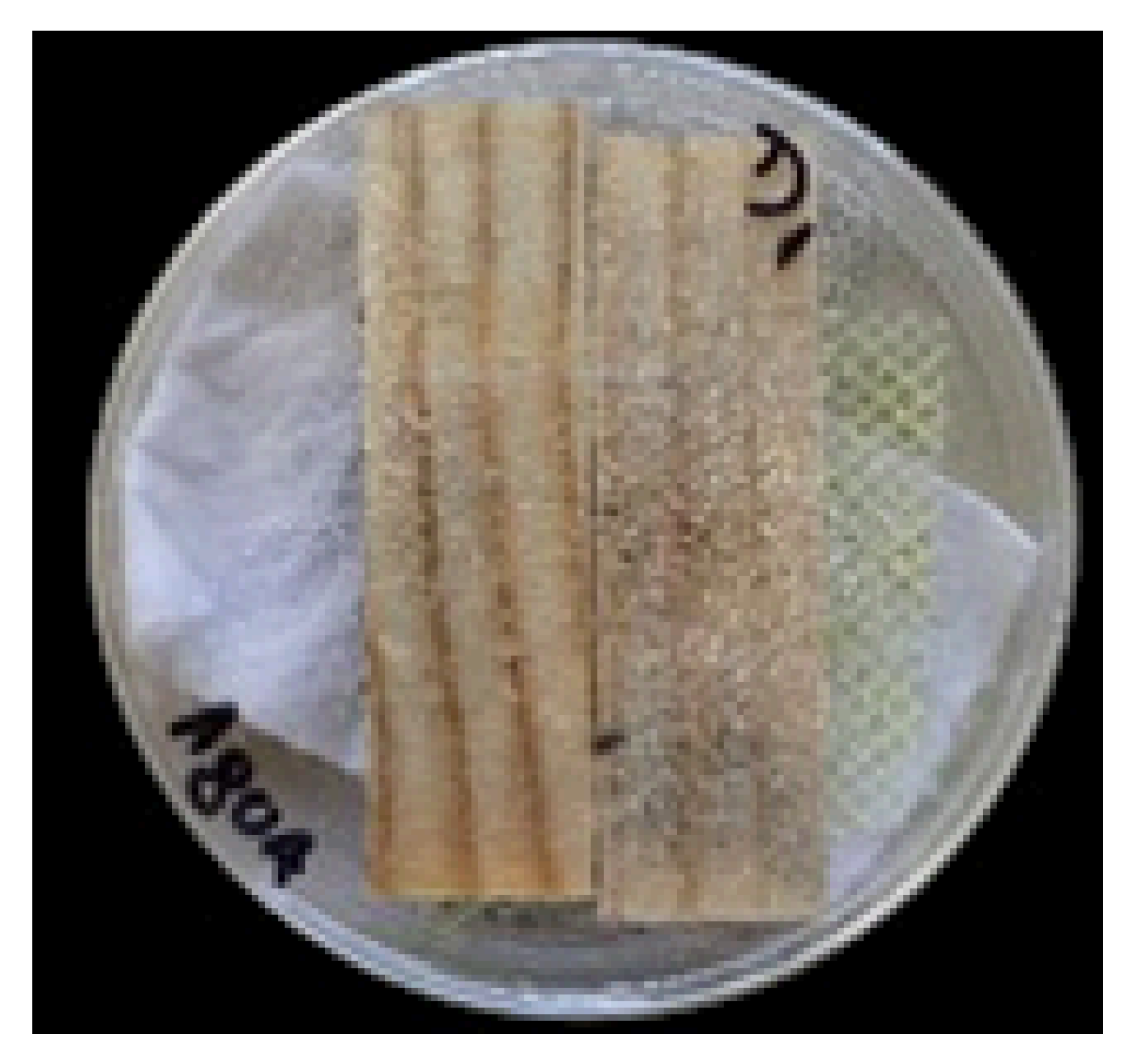
Nipacide P511	0	0	0	0 ^A^	100.00	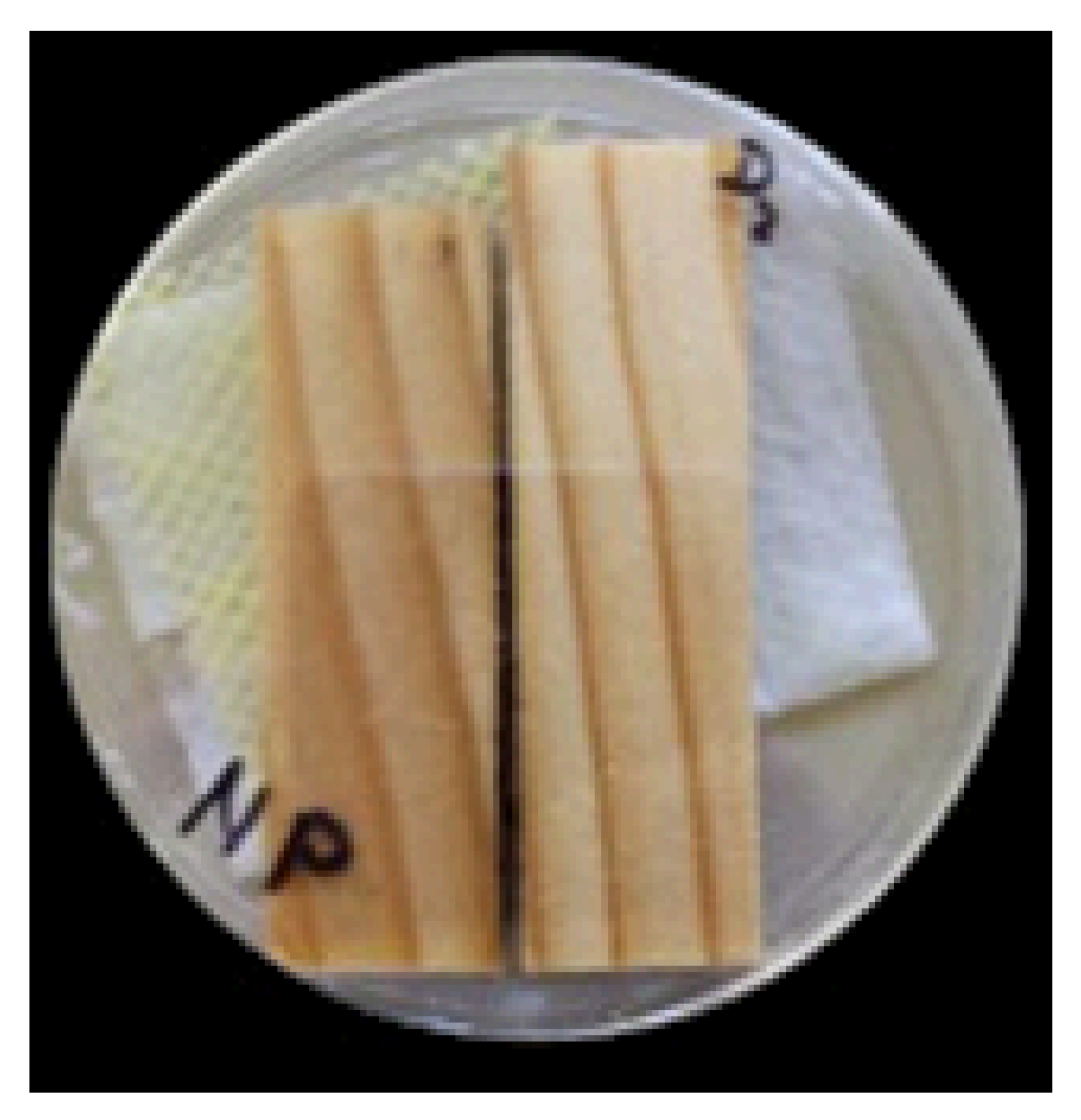

Values are expressed as mean ± standard deviation. Values in the same column with the same superscript are not significantly different from each other for each species. The wood specimens were immersed in each solution for 10 s, then aerated in a sterile environment for 24 h before inoculation with the fungi. Columns “Value 1–3” report the individual 0–5 visual ratings of the three replicates and not an assigned score; the corresponding mean is given in the “Mean ± SD” column. A score of 5 denotes full surface colonization. AE100L: Sample treated with the extract obtained by alkali-assisted extraction in the 100 L reactor (solution of 2.5% *w*/*w*); NaOH solution: Sample treated with a solution of 0.05% *w*/*v* NaOH; Water: Sample treated with water; Nipacide P511: Commercial product used solely for staining fungi assays, prepared as a 5% *v*/*v* aqueous solution.

**Table 6:** Inhibition percentage of the growth of the mold *T. viride*.

Treatment	Value 1	Value 2	Value 3	Mean ± SD	Inhibition (%)	Final Condition
AE100L	0	0	0	0 ^A^	100	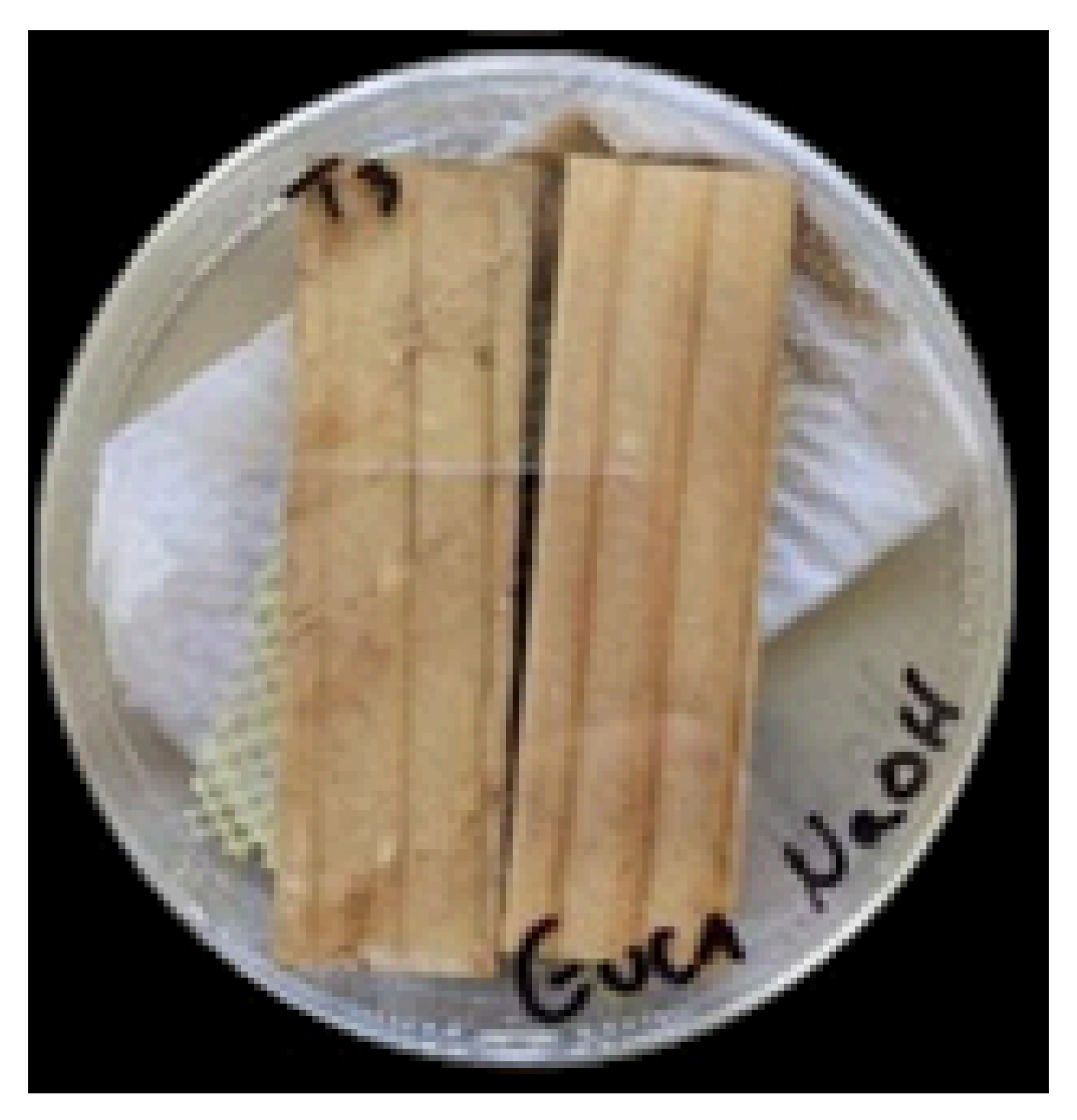
NaOH solution	5	5	5	5.00 ± 0.00 ^B^	0	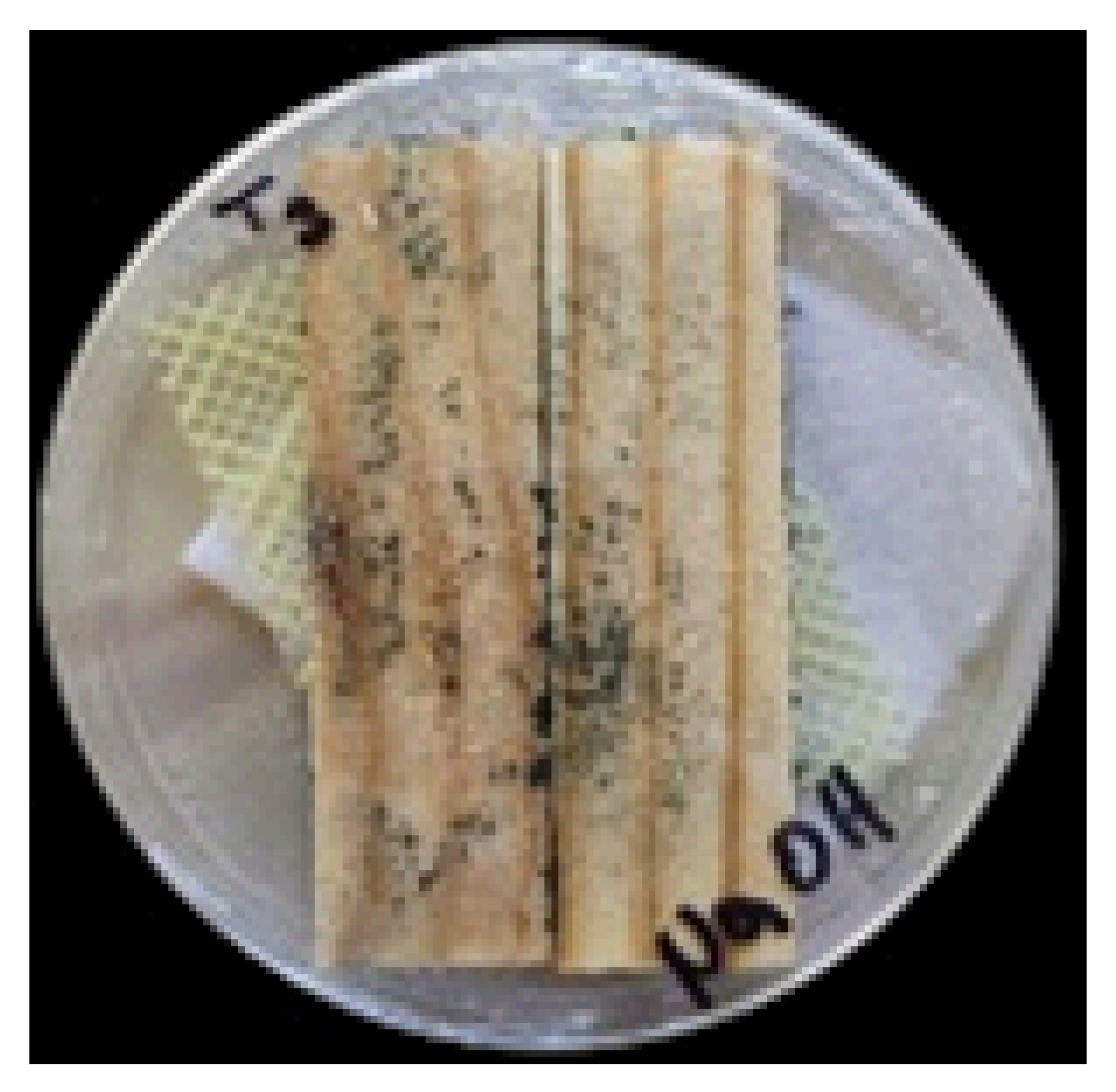
Water	5	5	5	5.00 ± 0.00 ^B^	0	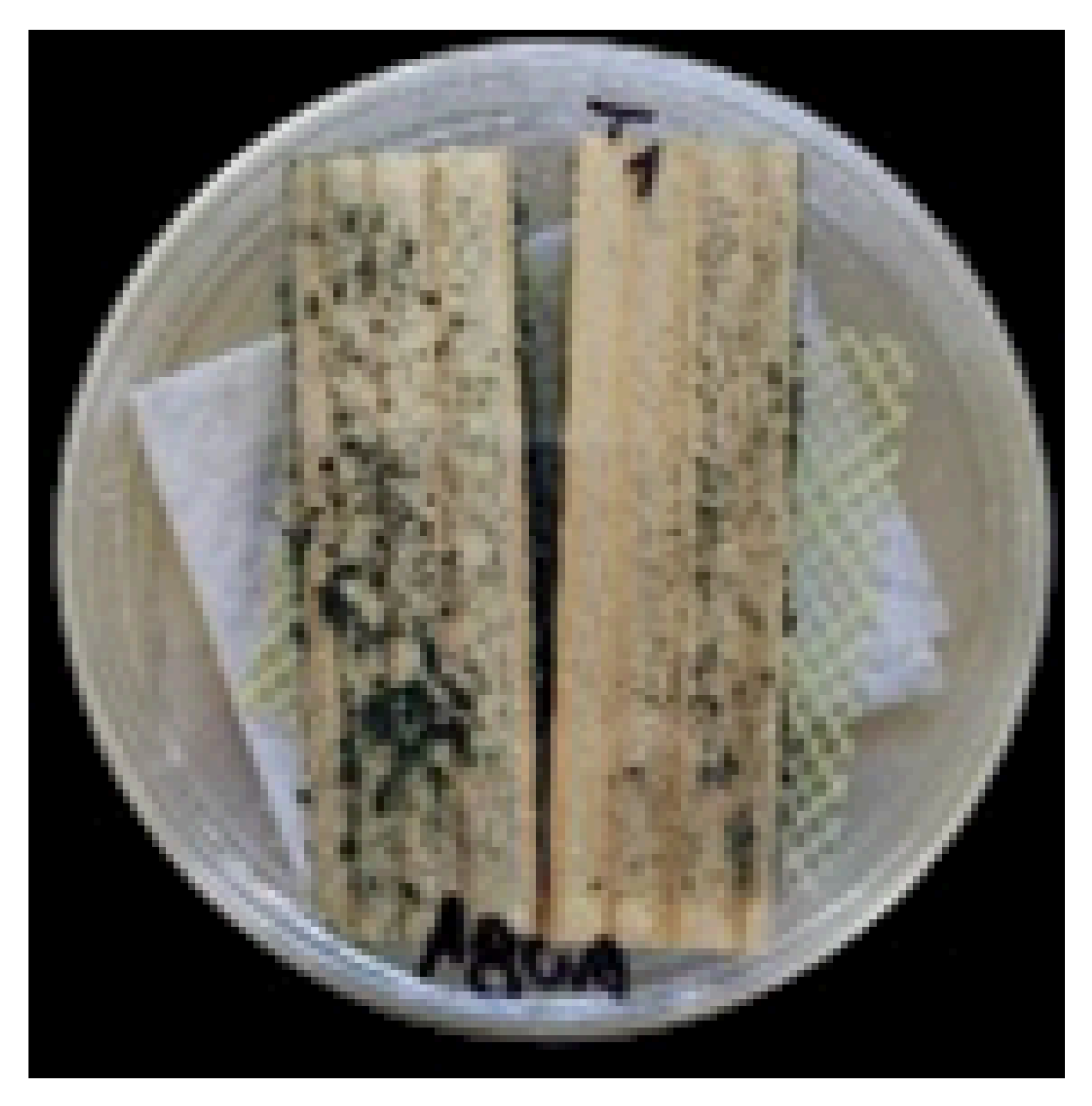
Nipacide P511	0	0	0	0 ^A^	100	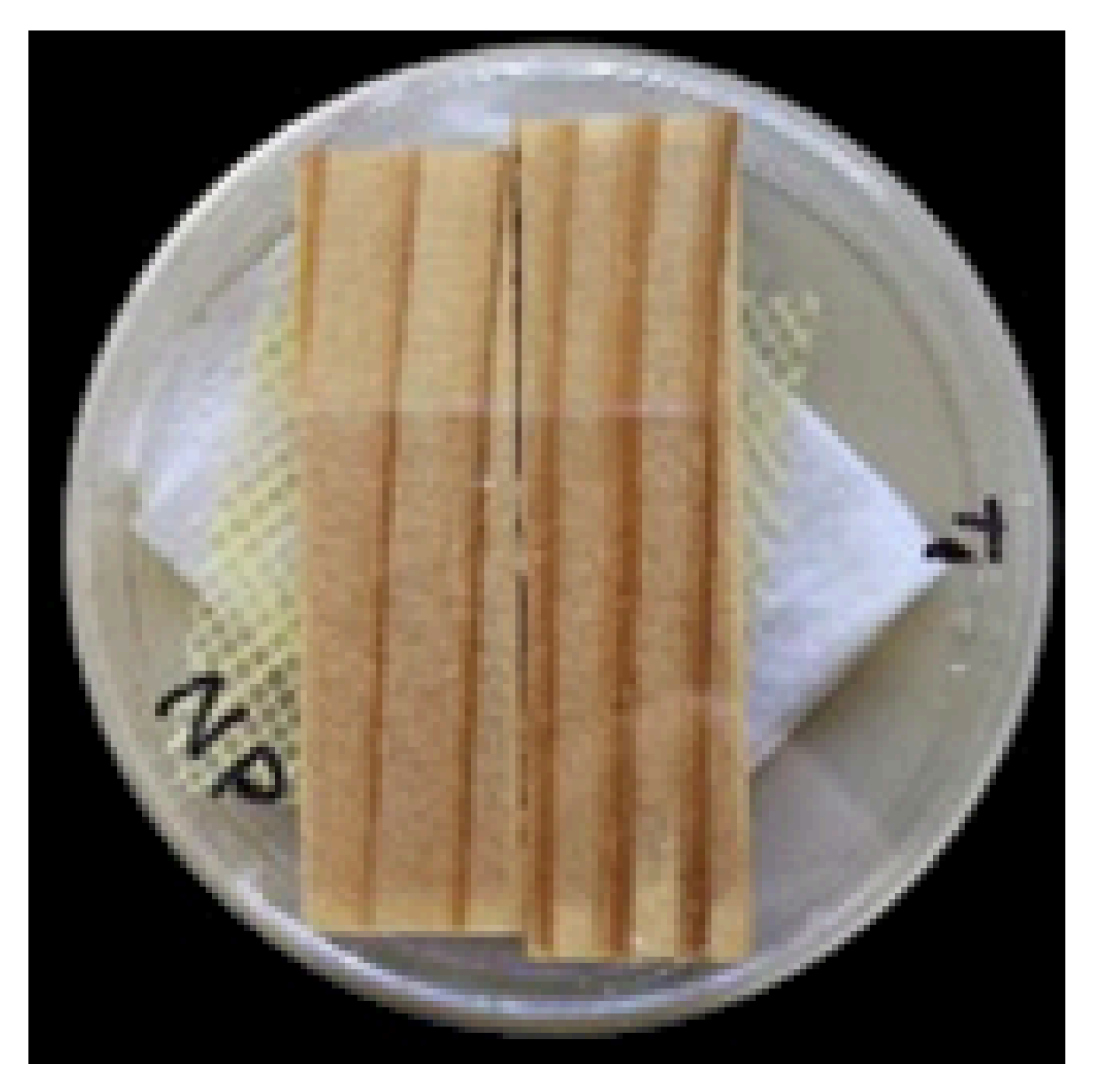

Values are expressed as mean ± standard deviation. Values in the same column with the same superscript are not significantly different from each other for each species. The wood specimens were immersed in each solution for 10 s, then aerated in a sterile environment for 24 h before inoculation with the fungi. Columns “Value 1–3” report the individual 0–5 visual ratings of the three replicates and not an assigned score; the corresponding mean is given in the “Mean ± SD” column. A score of 5 denotes full surface colonization. AE100L: Sample treated with the extract obtained by alkali-assisted extraction in the 100 L reactor (solution of 2.5% *w*/*w*); NaOH solution: Sample treated with a solution of 0.05% *w*/*v* NaOH; Water: Sample treated with water; Nipacide P511: Commercial product used solely for staining fungi assays, prepared as a 5% *v*/*v* aqueous solution.

**Table 7:** Weight loss (WLD%) after brown-rot fungus (*P. placenta*) decay test.

Treatment	WLD (%)	Final Condition
AE100L	−1.21 ± 0.48 ^A^	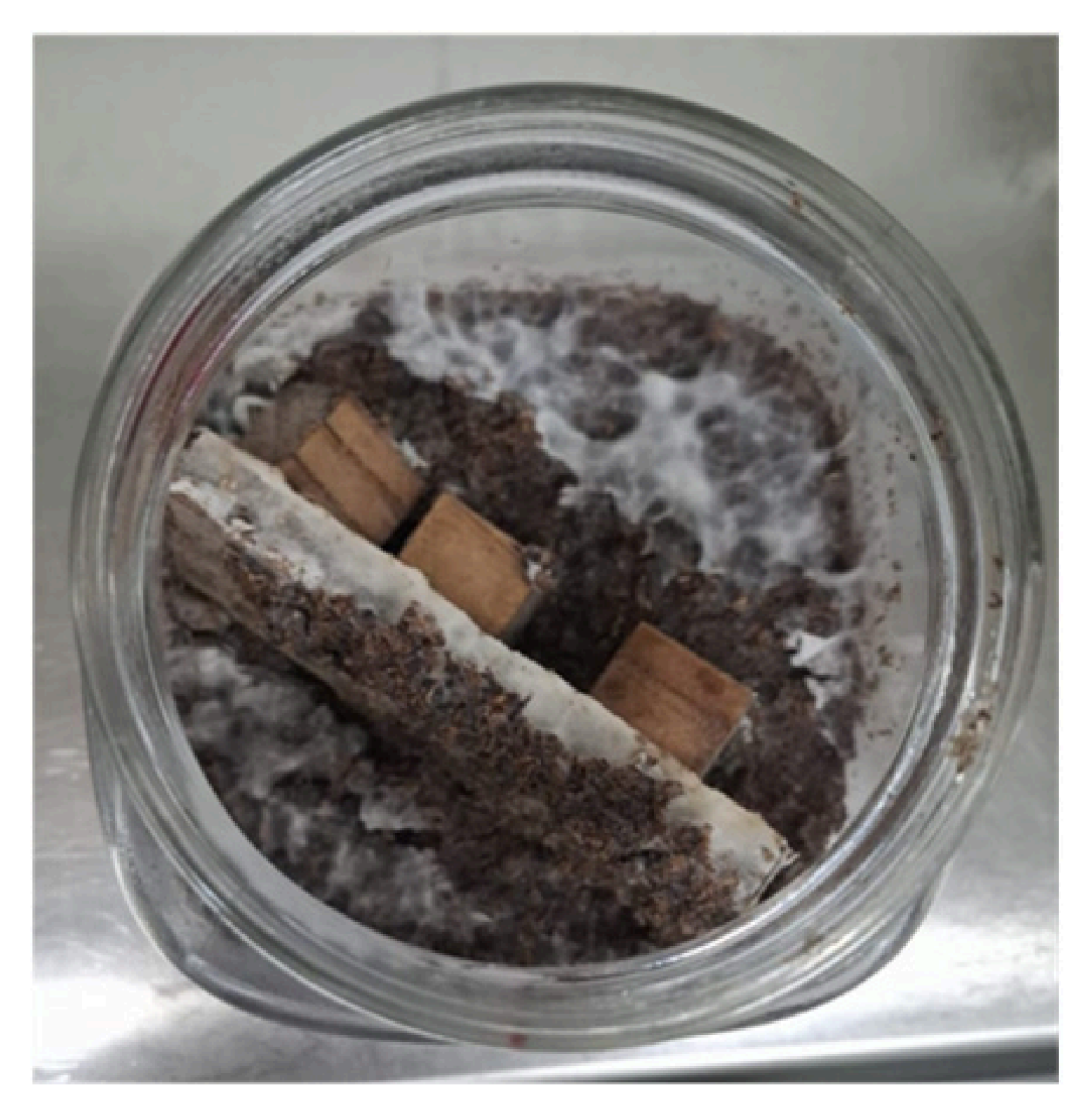
NaOH solution	46.13 ± 4.19 ^B^	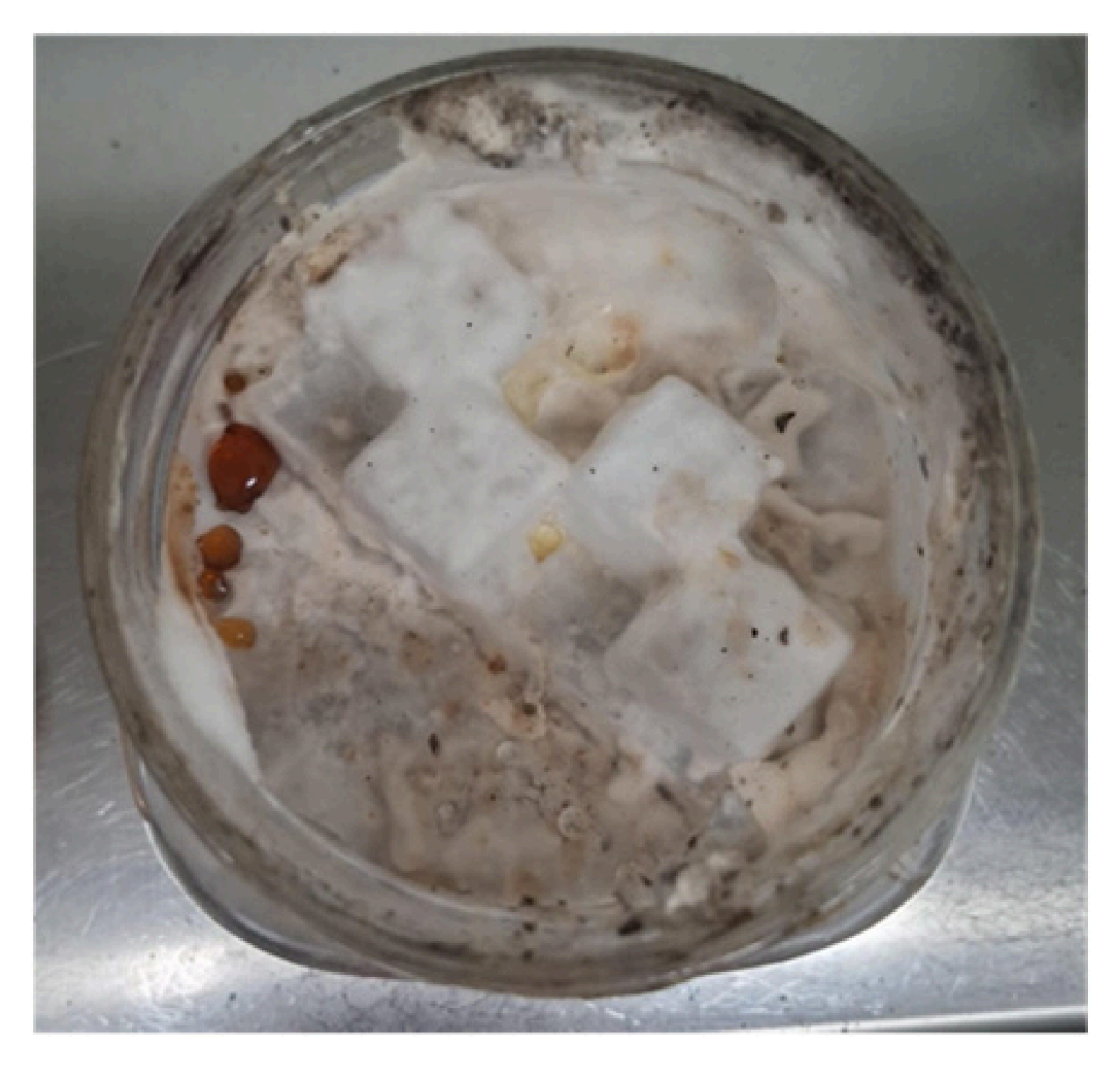
Water	53.16 ± 4.04 ^B^	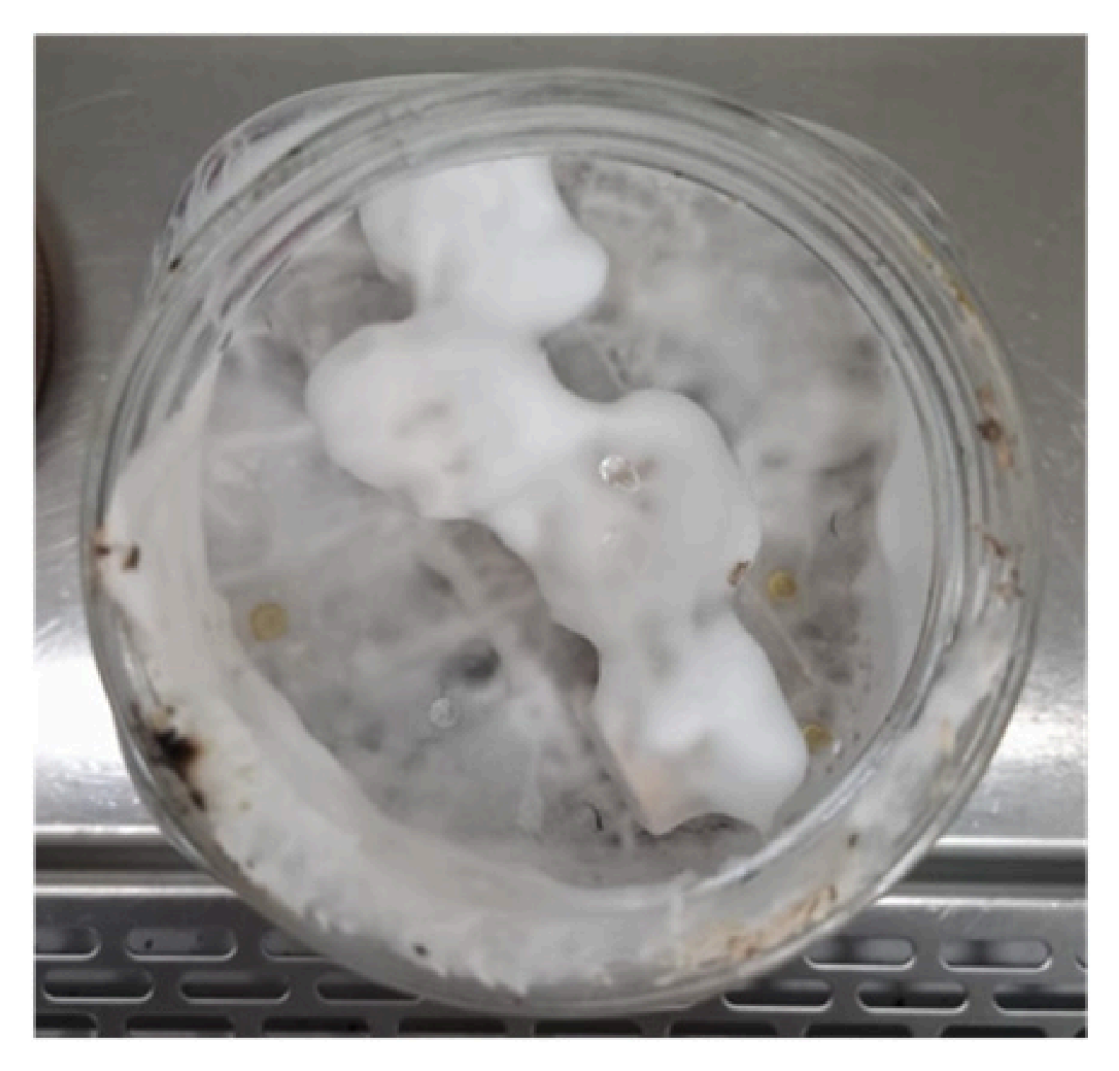

Values are expressed as mean ± standard deviation. Values in the same column with the same superscript are not significantly different from each other for each species. AE100L: Sample treated with the extract obtained by alkali-assisted extraction in the 100 L reactor (solution of 2.5% *w*/*w*); NaOH solution: Sample treated with a solution of 0.05% *w*/*v* NaOH; Water: Sample treated with water.

## Data Availability

The original contributions presented in this study are included in the article. Further inquiries can be directed to the corresponding author.

## Supplementary Materials

The following supporting information can be downloaded at: https://www.mdpi.com/article/10.3390/antiox15060774/s1. Figure S1: Total ion chromatogram (TIC) of the *E. globulus* bark alkaline extract, obtained by LC-ESI-LTQ-Orbitrap-MS in negative ion mode.
