# Supplementary material for: Pilot Alkaline Extraction of Eucalyptus globulus Bark: A Natural Sustainable Solution for Wood Preservation
Source: Antioxidants (Basel). 2026 Jun 22;15(6):774. doi: 10.3390/antiox15060774 (PMC13296115; doi:10.3390/antiox15060774)
Supplement: Supplementary file 1 [file antioxidants-15-00774-s001.zip › antioxidants-4349479-supplementary.pdf]

antioxidants

*Supplementary Materials*

**Figure S1.** Total Ion Chromatogram (TIC) of the *E. globulus* bark alkaline extract.

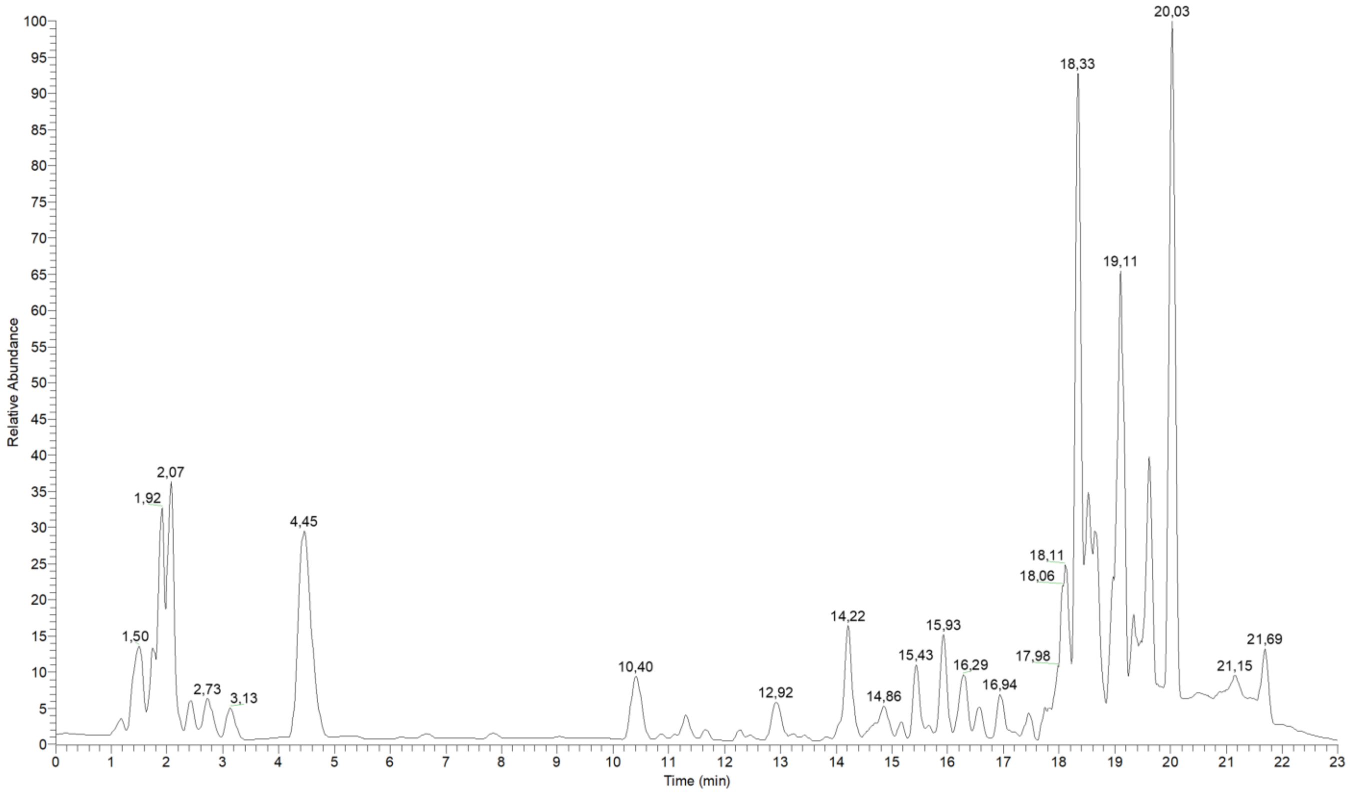


Total Ion Chromatogram (TIC) of the *E. globulus* bark alkaline extract, obtained by LC-ESI-LTQ-Orbitrap-MS in negative ion mode.
